# Mutant KRAS triggers functional reprogramming of tumor-associated macrophages in colorectal cancer

**DOI:** 10.1038/s41392-021-00534-2

**Published:** 2021-04-09

**Authors:** Huashan Liu, Zhenxing Liang, Chi Zhou, Ziwei Zeng, Fengwei Wang, Tuo Hu, Xiaowen He, Xiaojian Wu, Xianrui Wu, Ping Lan

**Affiliations:** 1 Department of Colorectal Surgery, The Sixth Affiliated Hospital, Sun Yat-sen University, Guangzhou, Guangdong China; 2 Guangdong Provincial Key Laboratory of Colorectal and Pelvic Floor Diseases, The Sixth Affiliated Hospital, Sun Yat-sen University, Guangzhou, Guangdong China; 3 Clinical Innovation Department, Guangzhou Regenerative Medicine and Health Guangdong Laboratory, Guangzhou, China; 4 Department of Colorectal Surgery, Sun Yat-sen University Cancer Center, Guangzhou, China; 5 State Key Laboratory of Oncology in South China, Collaborative Innovation Center for Cancer Medicine, Sun Yat-sen University Cancer Center, Guangzhou, Guangdong China

**Keywords:** Gastrointestinal cancer, Cancer microenvironment

## Abstract

Oncogenic KRAS has been previously identified to act in a cell-intrinsic manner to modulate multiple biological functions of colorectal cancer (CRC). Here, we demonstrate a cell-extrinsic role of KRAS, where KRAS engages with the tumor microenvironment by functional reprogramming of tumor-associated macrophages (TAMs). In human CRC specimens, mutant KRAS positively correlates with the presence of TAMs. Mutationally activated KRAS in tumor cells reprograms macrophages to a TAM-like phenotype via a combination effect of tumor-derived CSF2 and lactate. In turn, KRAS-reprogrammed macrophages were shown to not only promote tumor progression but also induce the resistance of tumor cells to cetuximab therapy. Mechanistically, KRAS drives the production of CSF2 and lactate in tumor cells by stabilizing hypoxia-inducible factor-1α (HIF-1α), a transcription factor that controls the expression of CSF2 and glycolytic genes. Mutant KRAS increased the production of reactive oxygen species, an inhibitor of prolyl hydroxylase activity which decreases HIF-1α hydroxylation, leading to enhanced HIF-1α stabilization. This cell-extrinsic mechanism awards KRAS a critical role in engineering a permissive microenvironment to promote tumor malignancy, and may present new insights on potential therapeutic defense strategies against mutant KRAS tumors.

## Introduction

Colorectal cancer (CRC) remains a frequently encountered fatal disease worldwide. Approximately 20% of CRC patients exhibit distant metastasis at the time of diagnosis.^1^ Individuals with metastatic CRC present with a median survival time of <2 years^2^ and a 5-year survival rate of 12–14%.^3^ Current therapies have been shown to offer only limited clinical benefit to metastatic CRC patients.^2,4^ Improved understanding of the molecular mechanisms underlying the pathogenesis of CRC is required to provide better therapeutic options and improve clinical outcomes of CRC patients.

KRAS mutation, which is typically associated with tumor progression and poor prognosis, was reported to occur in 35–50% of CRC patients.^5,6^ The roles of KRAS in CRC pathogenesis were faithfully indicated by genetically engineered CRC mouse models, in which the conditional expression of the mutant allele of KRAS promoted tumor invasion and metastases.^7^ Moreover, mutationally activated KRAS was found to elicit intrinsic resistance to epidermal growth factor receptor inhibitors.^8^ These insights imply that KRAS exerts complex effects in tumors.

In addition to the well-identified cell-intrinsic roles of KRAS, there is a growing recognition that KRAS acts via cell-extrinsic mechanisms to favor tumor progression by engaging with the tumor microenvironment.^9,10^ As a major component and key orchestrator of the tumor microenvironment, tumor-associated macrophages (TAMs) directly affect multiple tumor processes, including growth, invasion, metastasis, glycolysis, angiogenesis, and chemoresistance.^11–13^ TAMs are primarily recruited from peripheral blood monocyte-derived macrophages and/or tissue-resident macrophages.^14–16^ Then they are reprogrammed to acquire tumor-supportive capacity within the tumor microenvironment.^17,18^ However, the role of KRAS in triggering the pro-tumoral properties of TAMs has not been determined. Therefore, this study sought to specifically investigate the cell-extrinsic role of KRAS in tumor crosstalk with TAMs and the mechanism by which this occurs.

## Results

### KRAS mutation positively correlates with TAMs in CRC

**Fig. 1:**
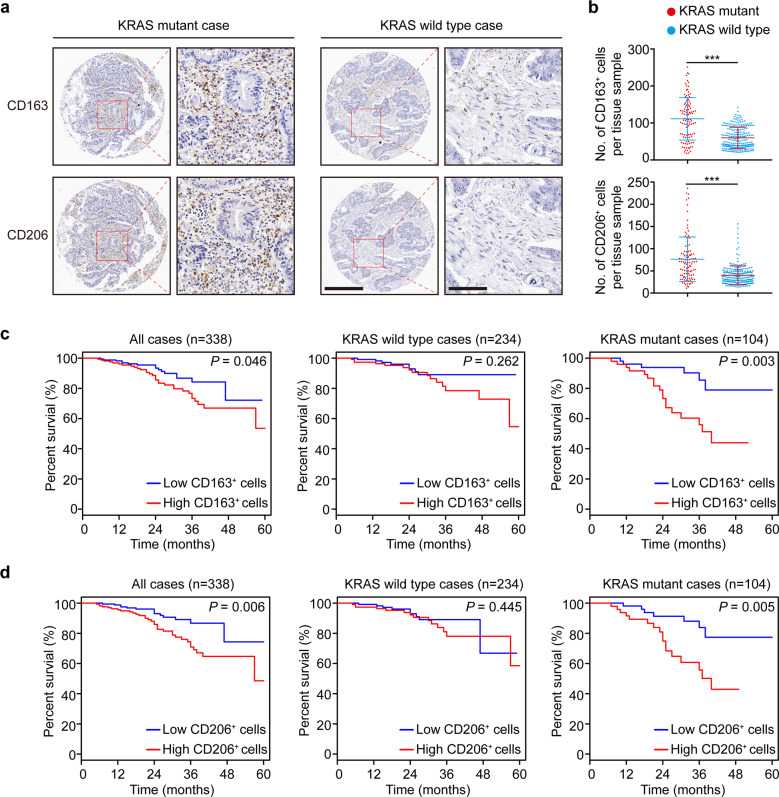
KRAS mutation positively correlates with TAMs in CRC. **a** Immunostaining of CD163 and CD206 in the tumor stroma in a representative human CRC case carrying mutant KRAS compared to a KRAS wild-type case. Scale bars: 400 μm (left), 100 μm (right). **b** Quantification of CD163^+^ and CD206^+^ macrophages in all 338 human CRC samples. **c**, **d** Kaplan–Meier survival curve of CRC patients layered by the density of CD163^+^ or CD206^+^ macrophages within the tumor stroma in tissue microarrays. ***p ≤ 0.001, by two-tailed Student’s *t*-test (**b**) or log-rank test (**c**, **d**)

To specifically probe the potential link between KRAS and TAMs in CRC, we examined the presence of TAMs in 338 CRC samples, including 104 KRAS mutant and 234 KRAS wild-type cases. Immunohistochemistry (IHC) was used to determine the TAM density as indicated by the CD163- and CD206-positive cells. The TAM density increased with more advanced TNM stages (Supplementary Table S1), an association which was also identified in subgroup analyses of patients with and without KRAS mutation (Supplementary Tables S2 and S3). Moreover, the TAM density was found to be more abundant in the primary tumors with KRAS mutation (Fig. 1a, b). The Kaplan–Meier curves showed that a high TAM density was associated with poor survival in all cases (CD163^+^ cells: log-rank test, *p* = 0.046; hazard ratio, 1.83; 95% CI, [1.00–3.37]; CD206^+^ cells: log-rank test, *p* = 0.006; hazard ratio, 2.34; 95% CI, [1.25–4.38]) (Fig. 1c, d). Stratification of the cohort into patients with (*n* = 104) and without (*n* = 234) KRAS mutation found that a high TAM density was associated with poor survival in KRAS mutant CRC cases (CD163^+^ cells: log-rank test: *p* = 0.003; hazard ratio, 3.63; 95% CI, [1.44–9.16]; CD206^+^ cells: log-rank test, *p* = 0.005; hazard ratio, 3.31; 95% CI, [1.37–8.01]), but not in cases with wild-type KRAS (log-rank test: *p* > 0.05) (Fig. 1c, d). These data suggest that there might be an interaction between TAMs and KRAS status in tumor cells which leads to a poor prognosis of CRC patients.

### KRAS mutant tumor cells reprogram macrophages to a TAM-like phenotype

**Fig. 2:**
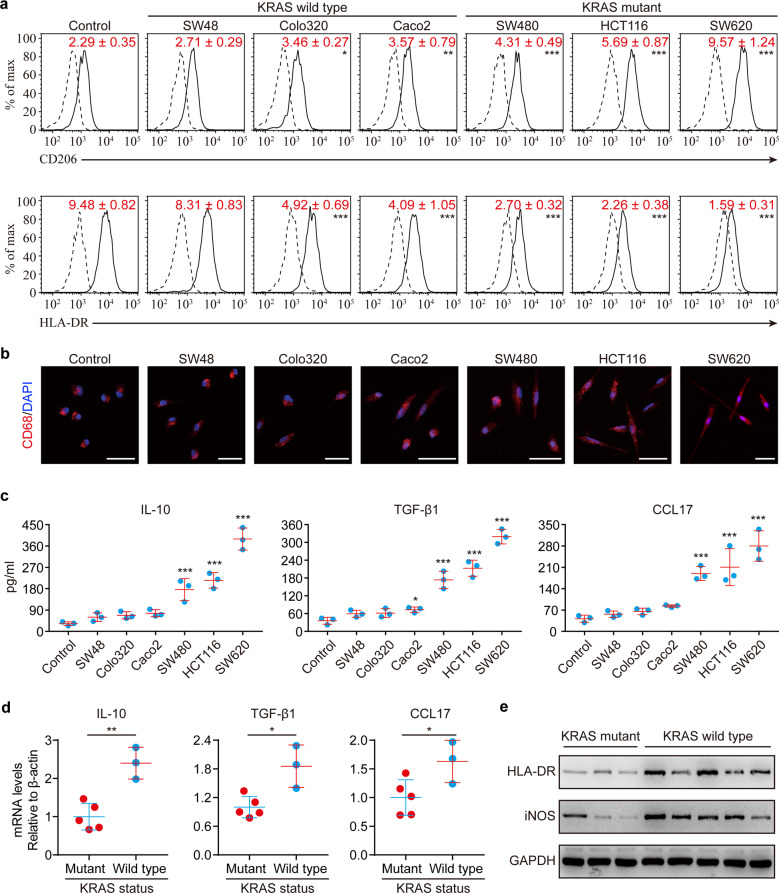
KRAS mutant tumor cells have a selective advantage to reprogram macrophages to a TAM-like phenotype. **a**–**c** Macrophages were obtained by culture of monocytes in DMEM medium supplemented with 10% heat-inactivated human AB serum in the presence or absence of 30% CM from the indicated cell lines for 6 days. **a** Representative flow cytometry staining for CD206/HLA-DR in macrophages. The solid lines represent cells stained with monoclonal antibodies, and dotted lines represent those stained with isotype controls. Numerical values denote the relative mean fluorescence intensity (RelMFI) normalized to isotype controls (mean ± SD) (*n* = 3). **b** Immunofluorescence images of CD68/DAPI in macrophages. Scale bars: 30 μm. **c** Cytokine levels in the media obtained from macrophage cultures (*n* = 3). **d**, **e** Primary TAMs were isolated from fresh CRC tumor samples. **d** The levels of indicated cytokines by real-time PCR assays and **e** the M1 makers by immunoblots in TAMs were compared between KRAS mutant CRC tissues (*n* = 3) and KRAS wild-type tissues (*n* = 5). *p*-values are for comparison with “Control” (**a**, **c**). *p ≤ 0.05, **p ≤ 0.01, and ***p ≤ 0.001, by one-way ANOVA (**a**, **c**) or by two-tailed Student’s *t*-test (**d**)

As demonstrated above, mutant KRAS was positively associated with the presence of TAMs. We, therefore, tested whether KRAS mutation in tumor cells has a role in the functional reprogramming of TAMs. Freshly isolated human monocytes were cultured in media with 30% conditioned medium (CM) from six CRC cell lines, including three lines with mutant KRAS (SW620, HCT116, SW480) and three with wild-type KRAS (Colo320, Caco2, SW48), for 6 days to obtain macrophages. Using the phenotype of primary TAMs as a reference,^19^ we found that CM from KRAS mutant lines, but not CM from KRAS wild-type lines, actively reprogramed macrophages to a TAM-like phenotype with a CD206^high^/HLA-DR^low^ expression, a stretched and elongated morphology, and increased production of tumor-supportive cytokines (Fig. 2a, c). Moreover, TAMs isolated from fresh CRC tissues were shown to have higher levels of IL-10, CCL17, and TGF- β1 in KRAS mutant patients as opposed to KRAS wild-type counterparts (Fig. 2d). In contrast, KRAS mutation was inversely correlated with an M1 phenotype (Fig. 2e).

To verify whether the selective advantage of tumor cells to reprogram TAMs is determined by their KRAS status, we disrupted KRAS signaling using a KRAS-specific siRNA pool in SW620 cells. This effectively abrogated the ability to induce TAM-like changes in macrophages (Supplementary Fig. 1a–c). Moreover, we also tested the effects of wild-type SW48 cells that had been stably transfected with cDNA encoding the KRAS^G12V^ mutation. CM from KRAS^G12V^-transfected SW48 cells was able to trigger a TAM-like phenotype in macrophages (Supplementary Fig. 1a–d). Similar findings were also observed for ectopic expression of KRAS^G12C^ and KRAS^G12D^ mutation in SW48 cells (Supplementary Fig. 1e, f), suggesting that the reprogramming of TAMs is unlikely to correlate with KRAS mutation type. Transfection of KRAS^G12V^ mutation in SW48 cells significantly increased the ability to reprogram macrophage, while transfection of wild-type KRAS only slightly increased the ability (Supplementary Fig. 1g). Furthermore, the effects elicited by transfection of KRAS^G12V^ mutation, rather than wild-type KRAS, seemed to be in a dose-dependent manner according to the KRAS expression levels (Supplementary Fig. 1g). Taken together, these findings suggest that mutationally activated KRAS in CRC cells directly elicits the functional reprogramming of TAMs.

### KRAS-reprogrammed macrophages exert tumor-supportive capacity

In light of our above findings, we subsequently explored the biological functions of KRAS-reprogrammed macrophages (KRAS-Mφ) in tumor progression. To address this, KRAS-Mφ were generated by culture of monocytes in DMEM medium in the presence of 30% CM obtained from KRAS mutant SW620 cells for 6 days. Macrophages untreated (Ut-Mφ), or treated with CM from SW620 cells that had been mock-transfected (siMOCK-Mφ), or transfected with KRAS-siRNAs (siKRAS-Mφ) were used as controls. Afterward, SW48 cells were cocultured with Ut-Mφ, KRAS-Mφ, siMOCK-Mφ, and siKRAS-Mφ in Transwell plates for 7 days and then collected for further experiments. Coculture with KRAS-Mφ significantly enhanced the tumor growth capacity, as determined by soft agar colony formation (Supplementary Fig. 2a, e) and plate colony formation (Supplementary Fig. 2b, f). SW48 cells cocultured with KRAS-Mφ exhibited a marked increase in migratory and invasive capacity (Supplementary Fig. 2c, d, g, h), and displayed decreased levels of E-cadherin but increased levels of Vimentin (Supplementary Fig. 2i). Importantly, SW48 cells cocultured with KRAS-Mφ displayed significantly increased aerobic glycolysis (Supplementary Fig. 2j–k), enhanced glucose consumption (Supplementary Fig. 2l) as well as lactate production (Supplementary Fig. 2m). Likewise, the correlation between KRAS and glycolysis was further reinforced by immunostaining analysis in CRC tissues, which revealed that tumors with mutant KRAS had a higher expression of tumor glycolytic enzymes, including GLUT1, GLUT3, and HK2, than the tumors with wild-type KRAS (Supplementary Fig. 2n, o). Collectively, these results indicate that KRAS-Mφ are able to elicit a significant increase of the aerobic glycolysis of tumor cells, which subsequently may result in enhanced proliferative and invasive capacities.

**Fig. 3:**
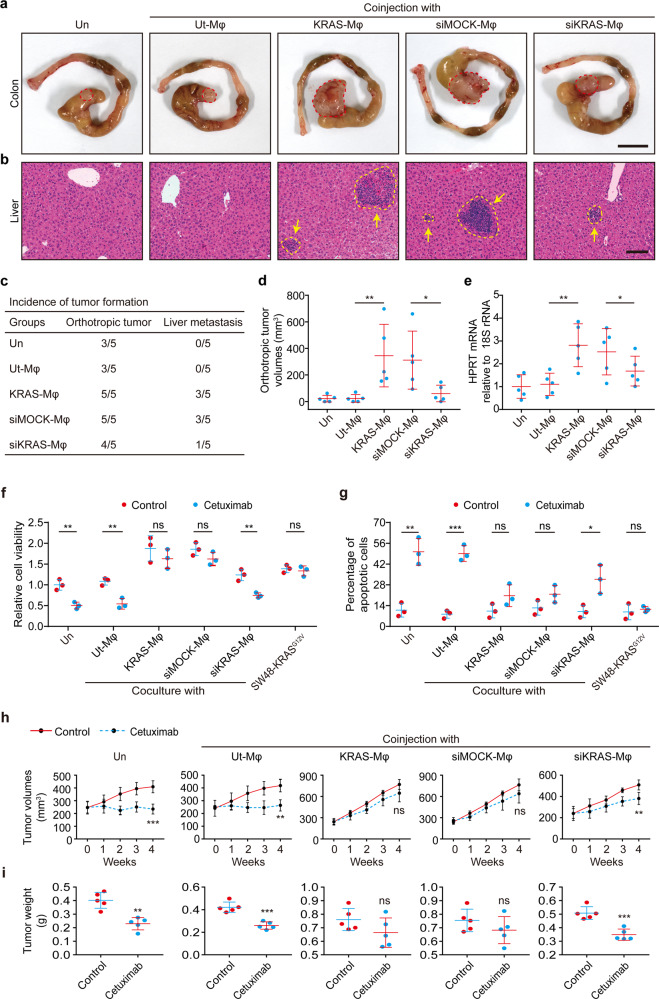
Effects of KRAS-reprogrammed macrophages on tumor cells. **a**–**i** KRAS-reprogrammed macrophages (KRAS-Mφ) were obtained by culture of monocytes in DMEM medium in the presence of 30% CM from KRAS mutant SW620 cells for 6 days. Untreated macrophages (Ut-Mφ) or those exposed to CM from SW620 cells that had been mock-transfected (siMOCK-Mφ), or transfected with KRAS-siRNAs (siKRAS-Mφ) were used as controls. **a**–**e** 2 × 10^6^ SW48 cells alone or mixed with 4 × 10^5^ Ut-Mφ, KRAS-Mφ, siMOCK-Mφ, or siKRAS-Mφ, were orthotopically injected into the wall of the cecum into 6-week old NOD-SCID mice (*n* = 5 mice per group). **a** Gross inspection of the CRC orthotopic tumors with each indicated treatment. **b** Representative H&E staining of liver micro-metastasis in mice xenografted as indicated. **c** Orthotopic xenograph CRC tumor formation and liver metastasis analysis. **d** Statistical analysis of tumor volumes and **e** expression levels of human HPRT mRNA relative to mouse 18 S rRNA in the livers of orthotropic xenograft CRC models as described above. **f**, **g** SW48 cells were cocultured with the indicated macrophages in Transwell systems for 7 days. Subsequently, the SW48 cells were treated with 0.2 μg/ml cetuximab or IgG control for 96 h and evaluated for proliferation by MTS staining (**f**), or for apoptosis by PI/Annexin V staining (**g**). **h**, **i** 5 × 10^6^ SW48 cells alone or mixed with 1 × 10^6^ Ut-Mφ, KRAS-Mφ, siMOCK-Mφ, or siKRAS-Mφ, were injected subcutaneously into 6-week old NOD-SCID mice. When tumors reached volumes of 200–300 mm^3^, mice bearing tumors were treated continuously by intraperitoneal injection with cetuximab or IgG control (1 mg twice a week; *n* = 5 mice per group). **h** Tumor growth curves during the course of each indicated treatment. **i** Tumor weights were measured after 4 weeks of cetuximab treatment in mice xenografted as indicated. Un, SW48 cells alone inoculated into mice (**a**–**e**, **h**, **i**) or SW48 cells without any treatment (**f**, **g**). Scale bars: 1.0 cm (**a**); 100 μm (**b**). *p ≤ 0.05, **p ≤ 0.01, ***p ≤ 0.001 and ns indicates *p* > 0.05, by one-way ANOVA (**d**, **e**) or by two-tailed Student’s *t*-test (**f**–**i**)

The in vivo function of KRAS-Mφ to CRC progression was determined through tumor xenograft models. First, we mixed KRAS-Mφ with SW48 cells, and then co-injected them subcutaneously as xenograft tumors into NOD-SCID mice. We found that co-injection of KRAS-Mφ markedly enhanced tumor growth, as shown by tumor size and weight (Supplementary Fig. 3a, b). These results were further supported by IHC results of CD163, Ki67, Vimentin, and GLUT1 in xenograft tumors (Supplementary Fig. 3c). We further tested the effects of KRAS-Mφ on tumor progression in the orthotopic xenograft tumor models. SW48 cells alone or mixed with the indicated macrophages were injected orthotopically into the wall of the cecum. After 8 weeks, 3 of the 5 mice (60%) injected with SW48 cells formed orthotopic tumors, but no liver metastasis was identified (Fig. 3a, c). In contrast, all of the 5 mice (100%) injected with SW48 cells mixed with KRAS-Mφ or siMOCK-Mφ exhibited orthotopic tumor formation (Fig. 3a, c), with larger tumor sizes (Fig. 3a, d). Moreover, CRC cells co-injected with KRAS-Mφ (3/5, 60%) or siMOCK-Mφ (3/5, 60%) were shown to be more likely to develop liver metastasis (Fig. 3b, c). H&E staining (Fig. 3b) along with quantification of human HPRT mRNA expression (Fig. 3e) confirmed that co-injection with KRAS-Mφ or siMOCK-Mφ could lead to a significant increase in the tumor burden of liver metastasis. Likewise, KRAS mutation status was found to be significantly associated with tumors’ T stage as well as lymph node and distant metastasis in CRC patients (Supplementary Table S4). Taken together, these findings suggest that the crosstalk between KRAS and TAMs may function as a previously unappreciated tumor-supportive mechanism in the tumor microenvironment.

### KRAS-reprogrammed macrophages contribute to the resistance of CRC to cetuximab

Previous work has established that CRC patients with mutant KRAS are lack of response to cetuximab therapy. We inquired whether the crosstalk between KRAS and TAMs contributes to this. A statistically significant cell growth inhibition was observed in SW48 cells after cetuximab treatment (Supplementary Fig. 4a). As anticipated, transfection of KRAS^G12V^ mutation into SW48 cells shifted them from cetuximab sensitive toward resistant (Supplementary Fig. 4a–c). We then cocultured cetuximab sensitive SW48 cells with the KRAS-Mφ and challenged the tumor cells with cetuximab. The survival of SW48 cells following cetuximab treatment was significantly enhanced upon coculture with KRAS-Mφ (Fig. 3f). In line with this, KRAS-Mφ effectively protected SW48 cells from cetuximab-induced apoptosis (Fig. 3g and Supplementary Fig. 4d). To verify the above findings in vivo, we employed a xenograft mouse model by co-injecting SW48 cells and KRAS-Mφ into NOD-SCID mice. Cetuximab significantly inhibited the growth of SW48 tumors compared to the control (Fig. 3h, i). More importantly, the inhibitory effects of cetuximab on tumor growth were attenuated when SW48 cells were co-injected with KRAS-Mφ or siMOCK-Mφ, but not Ut-Mφ or siKRAS-Mφ (Fig. 3h, i). Collectively, these data suggested that KRAS-Mφ contributed to the resistance of CRC to cetuximab.

### CSF2 synergizes with lactate to elicit functional reprogramming of TAMs

**Fig. 4:**
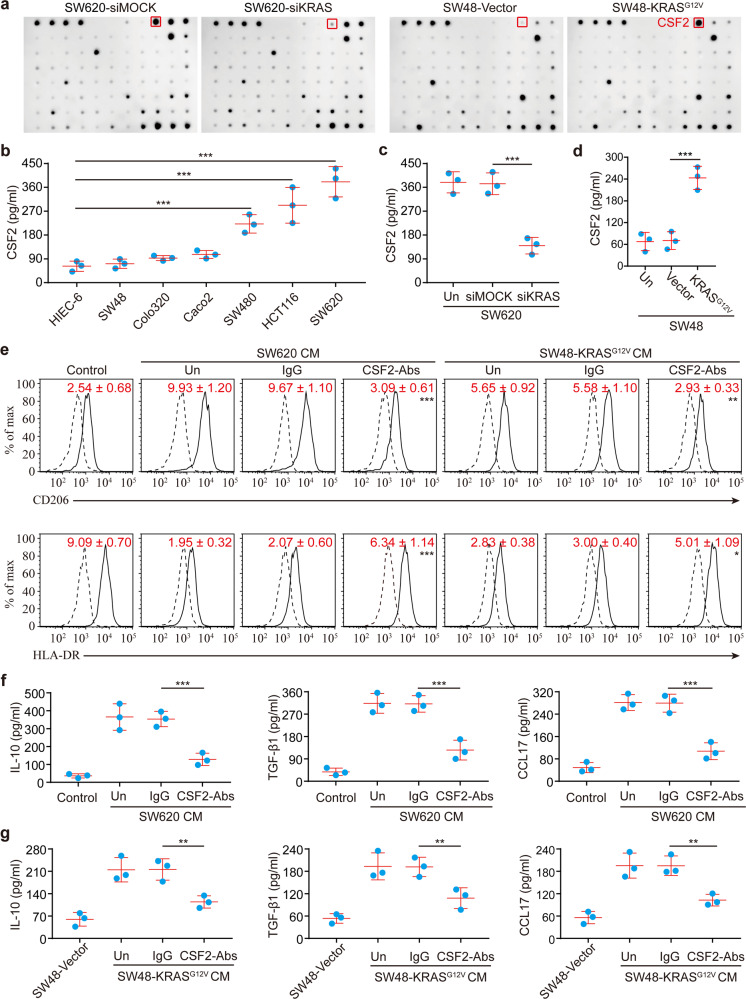
CSF2 is required for the macrophage reprogramming by KRAS mutant CRC cells. **a** Cytokine array of the CM of the indicated cells. CSF2 is denoted on the array by the red box. **b**–**d** The levels of CSF2 in the CM of the indicated CRC cells as determined by ELISA assays (*n* = 3). **e** Expression of CD206/HLA-DR in macrophages treated with 30% CM from the indicated tumor types as indicated in Fig. 2a–c in the presence or absence of control IgG or a CSF2 neutralizing antibody (*n* = 3). The solid lines represent cells stained with monoclonal antibodies, and dotted lines represent those stained with isotype controls. Numerical values denote the relative mean fluorescence intensity (RelMFI) normalized to isotype controls (mean ± SD). *p*-values are for comparison with “IgG”. **f**, **g** Cytokine levels in the media of macrophages treated as indicated in **e** (*n* = 3). *p ≤ 0.05, **p ≤ 0.01, and ***p ≤ 0.001, by one-way ANOVA (**b**–**g**)

As demonstrated above, we discovered a cell-extrinsic mechanism whereby oncogenic KRAS in tumor cells engages with TAMs. Subsequently, we sought to understand the mechanisms underlying the crosstalk between KRAS and TAMs. The cytokine profiles of CM from SW620 cells that were transfected with KRAS-siRNAs (SW620-siKRAS) or MOCK-siRNAs (SW620-siMOCK), and SW48 cells stably transfected with KRAS^G12V^ (SW48-KRAS^G12V^) or an empty vector (SW48-Vector) were delineated by RayBio Human Cytokine Antibody Array. Three cytokines, CSF2, G-CSF, and IL-7, were found to be substantially up-regulated in the CM derived from KRAS mutant cells (Fig. 4a and Supplementary Fig. 5a, b). ELISA assays further verified the significant increase of CSF2 in CM derived from KRAS mutant CRC cells (SW620, HCT116, SW480, SW48-KRAS^G12V^) compared to CM from KRAS wild-type cells (Colo320, Caco2, SW48, SW620-siKRAS) (Fig. 4b–d), whereas a similar pattern was not observed for G-CSF and IL-7 (Supplementary Fig. 5c). We, therefore, hypothesized that CSF2 may be a key player in KRAS mutant CRC cells that mediates the reprogramming of macrophages. As anticipated, the addition of a neutralizing anti-CSF2 antibody to the CM of SW620 cells significantly suppressed the induction of TAM-related surface marker expression and cytokines (Fig. 4e, f). A similar pattern was also observed for the CM from KRAS^G12V^-transfected SW48 cells (Fig. 4e, g). Ectopic expression of CSF2 in SW48 cells endowed them with the ability to reprogram macrophages (Supplementary Fig. 5e, f). Collectively, these results suggest that CSF2 is required for KRAS mutant CRC cells to reprogram macrophages to a TAM-like phenotype.

**Fig. 5:**
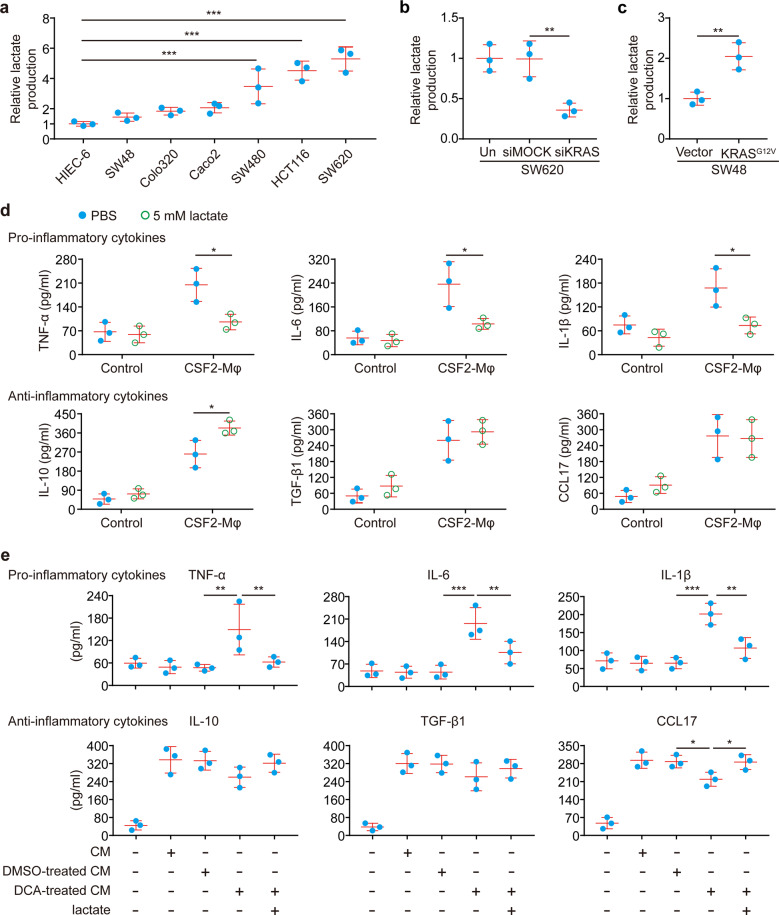
The pro-inflammatory activities of CSF2-activated macrophages are blunted by tumor-derived lactate. **a**–**c** Relative lactate production in the media of the indicated cells (*n* = 3). **d** Macrophages were obtained by culture of monocytes in culture medium in the presence or absence of 20 ng/ml CSF2 for 6 days. Afterward, macrophages were cultured in a culture medium with or without 5 mM lactate for 24 h. Cytokine levels in the media were measured by ELISA (*n* = 3 independent experiments using macrophages from three different donors). **e** SW620 cells were cultured in the presence or absence of 2 mM sodium dichloroacetate (DCA) for 72 h. CM was collected after the cells were cultured for another 24 h without DCA. In addition, the lactate level in CM obtained from SW620 cells treated with DCA was adjusted to the levels in untreated SW620 CM by the addition of lactate. Macrophages were obtained by culture of monocytes in culture medium in the presence or absence of the indicated SW620 CM for 6 days. The cytokine concentrations of the macrophages were measured afterward (*n* = 3 independent experiments using macrophages from three different donors). *p ≤ 0.05, **p ≤ 0.01, and ***p ≤ 0.001, by one-way ANOVA (**a**, **b**, **e**) or two-tailed Student’s *t*-test (**c**, **d**)

Subsequently, we asked whether CSF2 is sufficient to trigger functional reprogramming of TAMs. Importantly, we found that macrophages activated by exogenous CSF2 alone showed increased production of both pro-inflammatory and anti-inflammatory cytokines (Supplementary Fig. 5g, h). In contrast, tumor CM-activated macrophages only showed increased production of anti-inflammatory cytokines (Supplementary Fig. 5g, h), similar to TAMs. This implied that CSF2 maybe not the sole factor that contributes to tumor activation of macrophages, and there may exist other concomitant stimulation factors which abrogate the pro-inflammatory activities of CSF2-activated macrophages. Notably, tumor-derived lactate has been shown to contribute to a TAM-like phenotype.^20^ Moreover, we found that KRAS mutant CRC cells exhibited significantly elevated production of lactate relative to CRC cells with wild-type KRAS (Fig. 5a–c). These findings motivated us to test the effects of lactate on CM-activated macrophages. We found that administration of 5 mM lactate significantly blunted the production of pro-inflammatory cytokines (Fig. 5d), whereas it did not suppress the production of anti-inflammatory cytokines from CSF2-activated macrophages (Fig. 5d). The inhibition of pro-inflammatory cytokine production by SW620-derived CM was significantly blocked (Fig. 5e) when SW620 cells were pretreated with sodium dichloroacetate (DCA) to abolish the production of lactate. Furthermore, the CM obtained from DCA-pretreated SW620 cells acquired the ability to suppress the production of pro-inflammatory cytokines upon the addition of exogenous lactate (Fig. 5e). These findings suggest that tumor-derived lactate is responsible for the skew of CSF2-activated macrophages toward an anti-inflammatory state similar to TAMs. Taken together, we conclude that mutationally activated KRAS in tumor cells triggers the functional reprogramming of macrophages via a combination effect of CSF2 and lactate.

### Mutant KRAS drives the production of CSF2 and lactate via HIF-1α

As mentioned above, we demonstrated that CSF2 synergizes with lactate in the reprogramming macrophages by KRAS mutant cells. Subsequently, we inquired whether KRAS is directly responsible for the production of CSF2 and lactate in tumor cells. Disruption of KRAS signaling in KRAS mutant SW620 cells resulted in significantly attenuated production of CSF2 (Fig. 4c) and lactate (Fig. 5b). In addition, transfection of the KRAS^G12V^ mutation in KRAS wild-type SW48 cells led to increased production of CSF2 (Fig. 4d) and lactate (Fig. 5c). These findings suggested that mutationally activated KRAS drives the production of CSF2 and lactate by tumor cells.

**Fig. 6:**
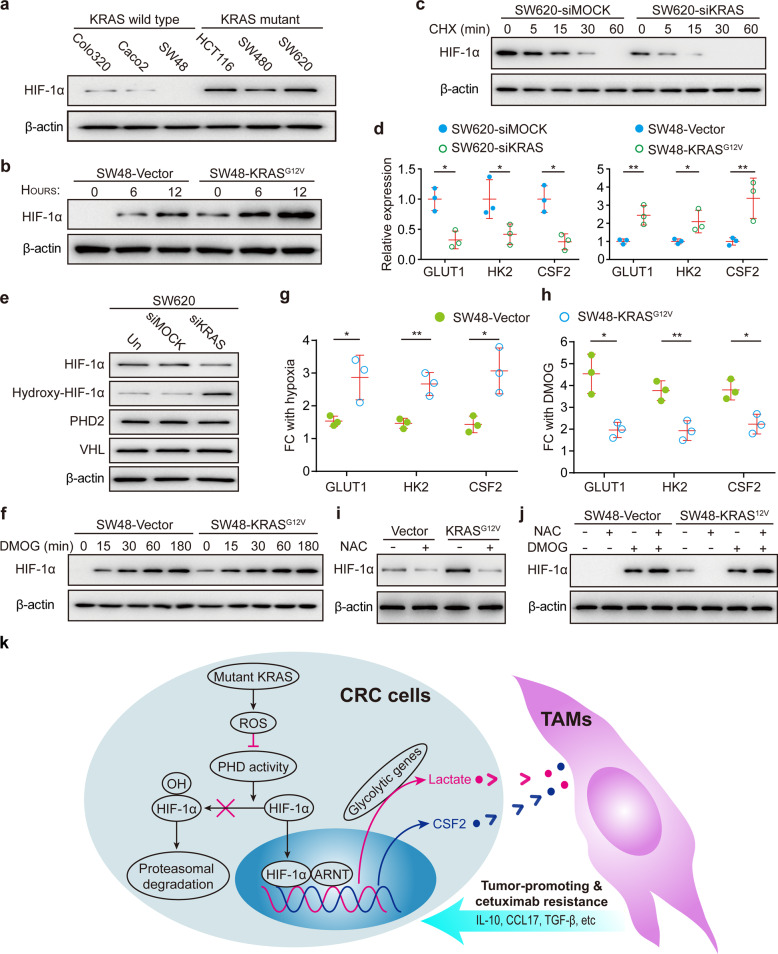
KRAS drives the production of CSF2 and lactate in tumor cells by stabilizing HIF-1α. **a** Representative immunoblot for HIF-1α in whole-cell lysates from KRAS mutant and wild-type CRC cells cultured at 21% O_2_. **b** Representative immunoblot for HIF-1α in SW48 cells expressing SW48-Vector or SW48-KRAS^G12V^ cultured at 1% O_2_ for the indicated times. **c** Representative immunoblot of the expression kinetics of HIF-1α in MOCK (SW620-siMOCK) or KRAS siRNA-transfected (SW620-siKRAS) SW620 cells treated with the transcription inhibitor cycloheximide (CHX, 100 μg/ml) for the indicated times at 21% O_2_. **d** HIF-1α target genes were measured by qRT-PCR (*n* = 3) in the indicated cells cultured under normoxia. **e** Representative immunoblot of the expression of HIF-1α, hydroxy-HIF-1α, PHD2, and VHL in MOCK or KRAS siRNA-transfected SW620 cells cultured at 21% O_2_. Before protein extraction, cells were treated with a proteasome inhibitor (10 μM MG-132) for 1 h to demonstrate hydroxyl-HIF-1α. **f** Immunoblots for HIF-1α in SW48-Vector or SW48-KRAS^G12V^ cells treated with 1 mM DMOG for the indicated times at 21% O_2_. **g** Fold change (FC) of HIF-1α target genes (GLUT1, HK2, CSF2) in response to hypoxia (*n* = 3) measured by qRT-PCR. The ratio of hypoxic to normoxic gene expression is shown. **h** FC of HIF-1α target genes in response to DMOG treatment was measured by qRT-PCR and the ratio of untreated to DMOG-treated gene expression is shown (*n* = 3). **i** Immunoblots of SW48-Vector or SW48-KRAS^G12V^ cells incubated with 10 mM N-acetylcysteine (NAC) and cultured at 1% O_2_. **j** Immunoblots of SW48-Vector or SW48-KRAS^G12V^ cells cultured at 21% O_2_ with 10 mM NAC or 1 mM DMOG as indicated. **k** Schematic summarizing our proposed model for the crosstalk between KRAS and TAMs in CRC. KRAS drives the production of CSF2 and lactate in tumor cells by stabilizing HIF-1α, and CSF2 synergizes with lactate to induce functional reprogramming of TAMs, which in turn supports tumor progression. β-actin served as loading controls and 3 independent experiments were performed and yielded similar results (**a**–**c**, **e**, **f**, **i**, **j**). β-actin serves as a housekeeping gene for qRT-PCR (**d**, **g**, **h**). *p ≤ 0.05 and **p ≤ 0.01, by two-tailed Student’s *t*-test (**d**, **g**, **h**)

Next, we tried to clarify the underlying mechanisms by which mutant KRAS drives the production of CSF2 and/or lactate. Notably, HIF-1α serves as the transcription factor that regulates the production of CSF2,^21^ as well as the primary driver of lactate production via increased glycolysis.^22,23^ We hypothesized that HIF-1α might be involved in the increased production of CSF2 and/or lactate driven by mutant KRAS. To test this, we first investigated whether KRAS directly regulates HIF-1α stability. We found that whole-cell lysates from KRAS mutant cells under normoxic conditions displayed significantly elevated levels of HIF-1α compared to lysates derived from cells with wild-type KRAS (Fig. 6a). HIF-1α was stabilized earlier and to a higher degree in KRAS^G12V^-transfected SW48 cells relative to SW48-Vector cells under hypoxic conditions (Fig. 6b). Disruption of KRAS signaling in SW620 cells using a KRAS-specific siRNA pool significantly shortened the half-life of HIF-1α protein during normoxia (Fig. 6c). Furthermore, the HIF-1α target genes (GLUT1, HK2, and CSF2) were significantly elevated in KRAS mutant cells (Fig. 6d). In addition, the positive correlation between mutant KRAS and HIF-1α expression was further confirmed by quantifying the staining of 24 CRC samples (Supplementary Fig. 6a, b). Together, these data demonstrate that KRAS contributed to the stabilization of HIF-1α and the induction of crucial HIF-1α target genes.

To examine the requirement for HIF-1α in the production of CSF2 and/or lactate in KRAS mutant cells, HIF-1α expression was silenced using a HIF-1α-specific siRNA pool. Silencing HIF-1α was found to significantly suppress the production of CSF2 and lactate in KRAS mutant tumor cells (Supplementary Fig. 6c, d), abrogating the ability to induce TAM-like changes in macrophages (Supplementary Fig. 6e, f). Importantly, the increase of CSF2 and lactate caused by stable transfection of KRAS^G12V^ in SW48 cells were effectively blunted upon silencing HIF-1α (Supplementary Fig. 6c, d). These data suggest that mutant KRAS drives the production of CSF2 and lactate through HIF-1α.

### KRAS-driven reactive oxygen species promotes the stabilization of HIF-1α

We next sought to understand how KRAS regulates HIF-1α stability. Under physiological conditions, HIF-1α is hydroxylated by prolyl hydroxylases (PHDs) and undergoes ubiquitin-mediated proteasomal degradation via von Hippel–Lindau (VHL) binding. Although disruption of KRAS signaling markedly reduced the levels of HIF-1α in SW620 cells, it significantly enhanced the extent of HIF-1α hydroxylation (Fig. 6e), indicating that PHD activity may mediate the elevated HIF-1α stability conferred by KRAS. To confirm this, we tested PHD activity in vector and KRAS^G12V^-transfected SW48 cells by treating cells with DMOG, a potent inhibitor of PHDs. In response to DMOG treatment, comparable levels of HIF-1α were observed in vector and KRAS^G12V^-transfected SW48 cells (Fig. 6f), suggesting that the presence of DMOG abrogates the differences in HIF-1α caused by KRAS, and that KRAS acts at the level of PHDs. Similar to the effects of hypoxia (Fig. 6g), DMOG enhanced the transcription of HIF-1α target genes (GLUT1, HK2, and CSF2) in both vector and KRAS^G12V^-transfected SW48 cells (Fig. 6h), further indicating that PHDs serve as the point of regulation by KRAS. KRAS^G12V^-transfected SW48 cells displayed a stronger response to hypoxia relative to vector cells (Fig. 6g), whereas transfection of KRAS^G12V^ significantly repressed the induction of HIF-1α target genes in response to DMOG treatment (Fig. 6h). These findings imply that PHD activity is already decreased in KRAS mutant cells. As a consequence, blockage of PHD activity by DMOG in KRAS mutant cells triggers a smaller change in PHD activity and thus a smaller induction of HIF-1α target genes. Together, these data point to a reduction of PHD activity leading to increased HIF-1α expression in KRAS mutant cells.

Current research indicates that multiple signal events perform key functions to affect PHD activity. Of note, reactive oxygen species (ROS) have been reported to inhibit PHDs and stabilize HIF-1α.^24,25^ Moreover, the production of ROS induced by hypoxia is essential to the hypoxic activation of HIF-1α.^26^ Given that KRAS mutant cells showed markedly elevated levels of ROS compared to cells with wild-type KRAS (Supplementary Fig. 6g), we hypothesized that increased ROS in KRAS mutant cells would contribute to the inhibition of PHD activity. First, we determined whether mutant KRAS could amplify the hypoxia-induced increase of ROS. We observed an increase in hypoxia-induced ROS levels in KRAS mutant cells (Supplementary Fig. 6h), suggesting a possible cause for the exaggerated response to hypoxia displayed by KRAS mutant cells. Next, the anti-oxidant N-acetylcysteine (NAC) was employed to inhibit the production of ROS. As anticipated, NAC reduced the levels of HIF-1α to similar levels in vector and KRAS^G12V^-transfected SW48 cells (Fig. 6i). In contrast, vector and KRAS^G12V^-transfected SW48 cells have comparable levels of HIF-1α induced by DMOG, and NAC was no longer able to destabilize HIF-1α in the presence of DMOG (Fig. 6j). Finally, we found that the increased expression of HIF-1α target genes caused by transfection of KRAS^G12V^ was effectively blocked after NAC treatment (Supplementary Fig. 6i). Taken together, we conclude that increased ROS triggered by mutant KRAS promotes HIF-1α stabilization (Fig. 6k).

## Discussion

The cell-intrinsic mechanisms by which KRAS performs key functions in various aspects of tumor biology are well-documented. Recently a cell-extrinsic role of mutant KRAS in the modification of the tumor microenvironment has come into focus.^9^ The key findings of our current study present important perspectives on the cell-extrinsic role of KRAS in the context of CRC progression (Fig. 6k). We found that CRC cells harboring mutant KRAS have a selective advantage to actively reprogram macrophages to a TAM-like phenotype, which in turn induces resistance of the tumor cells to cetuximab and promotes malignant progression. Mechanistically, mutationally activated KRAS stabilizes HIF-1α, which drives the production of CSF2 and lactate in tumor cells. Then CSF2 synergizes with lactate to induce functional reprogramming of TAMs. The presence of ROS in KRAS mutant cells contributes to increased HIF-1α stabilization. Identification of such interactions between KRAS and TAMs provides a better understanding of the mechanisms by which KRAS affects CRC pathogenesis, and might provide novel insights on potential therapeutic strategies for treating KRAS mutant tumors.

The cell-autonomous mechanisms of KRAS indicate that its mutational activation can affect many tumor cellular processes within the tumor cells themselves, such as cell cycle, apoptosis, cell junctions, growth, self-renewal, and metabolic reprogramming.^27–29^ An association between KRAS and TAMs in cancers has been established by pioneering investigations.^30,31^ In agreement with these previous studies, our findings demonstrate that KRAS can induce functional reprogramming of TAMs in the context of CRC. Furthermore, the reprogramming of TAMs is unlikely to correlate with KRAS mutation type. Cetuximab has been shown to provide clinical benefits to CRC patients with wild-type KRAS, however, those with mutant KRAS often fail to respond. The most straightforward mechanism of resistance to cetuximab is attributed to the influences of KRAS on tumor cells. This study showed that KRAS-Mφ can induce tumor cells’ resistance to cetuximab, highlighting a previously unappreciated role of KRAS in driving cetuximab resistance. These findings are novel and clinically relevant since it presents new insights into the effects of KRAS and extends our understanding of the resistance to cetuximab in CRC patients. In addition to its effects on macrophages, KRAS has been previously shown to play other vital roles in the formation of the tumor microenvironment, including recruiting of myeloid-derived suppressor cells^9^ and converting of conventional T cells into regulatory T cells.^10^ These insights confirm a definitive link between KRAS and the tumor microenvironment.

Emerging evidence suggests that tumor cells can actively communicate with TAMs to optimize the microenvironment for tumor progression in several cancers.^32^ TAM-featured inflammation has emerged as a hallmark of cancer.^33,34^ However, the association between TAMs density and patients’ clinical outcomes remains conflicting.^35^ Shimura demonstrated that the extent of TAM infiltration in human prostate cancer is inversely-associated with the clinical stage and reduced infiltration of TAMs is an independent predictor for time to disease progression.^36^ In contrast, Bronkhorst and colleagues^37^ showed that infiltration of TAMs gives a worse prognosis in uveal melanoma. In this study, we demonstrated that the TAM density has a negative impact on the prognosis of CRC patients. Further analyses indicated that the KRAS status seemed to matter when evaluating the association between TAM density and CRC patients’ survival since stratification of the cohort according to KRAS status showed that high TAM density predicted poor survival in KRAS mutant, but not in KRAS wild-type, CRC patients. These findings imply that KRAS has a critical role in the interactions between CRC cells and the microenvironment, granting these patients a poor prognosis.

To understand the mechanism by which KRAS engages with TAMs, we focused on the effects of KRAS on tumor cytokine profiles. We found that mutant KRAS altered the secretory cytokine profiles, revealing a previously underappreciated role of KRAS. Among the cytokines driven by mutational KRAS activation, we show that mutant KRAS causes a substantial increase in CSF2 production in several CRC cell lines, and that CSF2 is indispensable for the functional activation of TAMs. Our results indicate CSF2 may represent a mediator between KRAS and TAMs. Of note, there is a possibility that other factors induced by KRAS may synergize with each other to engineer permissive microenvironmental conditions for tumor growth and metastasis.

Our findings here also provided evidence for a tumor-supportive role of CSF2. However, previous studies have yielded contradictory results regarding the effects of CSF2 on tumor malignancy progression.^38^ The suppressive effect of CSF2 on tumor progression was supported by data showing that CSF2 can induce a protective immune response, which has led to its use as adjuvant tumor therapies.^39^ In contrast, some data show that CSF2-based anti-tumor therapy does not provide benefits to cancer patients, and can even lead to worse survival, directly challenging the tumor-suppressive role of CSF2.^40^ In accordance with this, endogenous CSF2 produced by tumor cells can elicit a tumor-supportive immune response,^41^ and is positively linked with tumor invasion and metastasis.^42^ Our results lend further support to this stimulatory role of CSF2 on tumor progression by showing that CSF2 contributes to the functional activation of TAMs, thereby promoting malignant phenotypes in CRC.

CSF2-activated macrophages have been shown to exhibit pro-inflammatory or anti-inflammatory activities in response to different concomitant stimulation factors.^43,44^ Su showed that lipopolysaccharide (LPS) can skew CSF2-activated macrophages to a pro-inflammatory phenotype.^19^ However, LPS seems unlikely to perform certain functions in KRAS-mediated functional reprogramming of TAMs because it is not commonly seen in the tumor microenvironment.^19^ Importantly, we observed that KRAS-reprogrammed macrophages are in an anti-inflammatory state, implying that there may exist some concomitant stimulation factors that abrogate the pro-inflammatory activities of CSF2-activated macrophages. We were able to identify lactate as this factor. In addition, we show that cancer cells harboring mutant KRAS have an advantage in lactate production. These data are consistent with the notion that KRAS-activating mutations disrupt cellular metabolism.^29^ Therefore, we concluded that lactate, one of the most abundant products of glycolysis, synergizes with CSF2 to reprogram macrophages to a TAM-like phenotype. Lactate may emerge as a widespread cofactor in the microenvironment of these tumors with mutant KRAS. Despite the evidence in this study, we do not rule out the possibility that other signaling events may participate in the induction of a TAM-like phenotype. In particular, further exploration is required to determine whether other stromal or immune cell types may also be involved in such an interplay.

Given its role in tumor pathogenesis, KRAS represents a viable target for therapeutics in human cancers. However, despite intensive efforts, strategies to target KRAS have been limited by the lack of a proper binding pocket for small molecules, and no applicable targeted strategy directly targeting KRAS itself or its downstream effectors has been approved for clinical use.^45,46^ A growing body of evidence implies that KRAS-driven malignancies are associated with extensive stromal remodeling which may be alternatively targeted to prevent tumor progression.^47,48^ In agreement, we here demonstrated that mutant KRAS induces an immune-suppressive microenvironment that favors tumor progression. Our results will be helpful in developing more effective therapeutic options that may benefit patients with KRAS mutant tumors. For instance, we showed that CSF2 synergizes with lactate to serve as immune modulators through the activation of TAMs, suggesting that both could serve as functional and translatable targets. As such, targeting CSF2 and/or lactate may provide a feasible route to reverse tumor-mediated immune suppression and augment the clinical effects of tumor immunotherapy. Given the importance of the ROS/PHD/HIF-1α axis in KRAS-driven production of CSF2 and lactate, inhibition of this axis alone or in combination with other immune modulators may emerge as more specific and refined options to further sensitize tumors to immunotherapy.

In summary, we discovered a cell-extrinsic mechanism of KRAS involving engagement with TAMs to induce the resistance of the tumor cells to cetuximab and promote cancer progression. These findings exert a potential for clinical applications, in which targeting KRAS and/or its downstream players might represent a novel approach to improve the outcomes of immunotherapy to CRC patients.

## Materials and methods

Detailed procedures are provided in Supplementary Experimental Procedures.

### Patients and tissue samples

Formalin-fixed, paraffin-embedded (FFPE) CRC tissues were obtained from 338 patients (104 KRAS mutant cases and 234 KRAS wild-type cases) who had undergone an operation at the Sixth Affiliated Hospital of Sun Yat-sen University. The procedures for related specimen collections were performed with the approval of the Institutional Review Board of the Sixth Affiliated Hospital of Sun Yat-sen University, and informed written consent was obtained from all subjects.

### Cell culture and treatment

Human CRC cell lines SW620, HCT116, SW480, Colo320, Caco2, SW48, and HIEC-6 were obtained from the American Type Culture Collection (ATCC). Cells were incubated at 37 °C in a 5% CO_2_-humidified incubator and cultured in DMEM medium (Gibco, NY, USA) or RPMI 1640 (Gibco, NY, USA) with 10% fetal bovine serum (Gibco, NY, USA) and 1% penicillin-streptomycin (Gibco, CA, USA). After growing to 80% confluence, cells were washed with PBS, and fresh serum-free media was added. Conditioned medium (CM) was harvested 48 h later, filtered through a 0.22-μm filter to remove cell debris, and then stored at −80 °C until use. To block the production of lactate, the indicated cells were treated with 2 mM sodium dichloroacetate (DCA, Sigma) for 72 h.

### Monocyte isolation and macrophage culture

Human peripheral blood mononuclear cells (PBMCs) were isolated using Ficoll density gradient (GE Healthcare, Buckinghamshire, UK) from healthy volunteer donors. The CD14^+^ monocytes from PBMCs were purified using a CD14^+^ Cell Isolation Kit (Miltenyi Biotec) based on the manufacturer’s instructions. Isolated cells were seeded at a density of 2 × 10^6^ cells/well in a 24-well plate in DMEM medium (GIBCO) with 10% heat-inactivated human AB serum (Gemini Bio-Products, West Sacramento, CA), 50 U/ml penicillin, 50 μg/ml streptomycin and 2 mM L-glutamine with or without 30% CM from various CRC cell lines or recombinant human 20 ng/ml CSF2 for 6 days.

### Coculture procedure

A 12-well Transwell plate with a 0.4-μm pore (Corning, Lowell, MA, USA) was employed for co-culturing of CRC cells and macrophages. CRC cells (5 × 10^5^) were added to the lower chamber, while macrophages (5 × 10^5^) were added to the upper chamber. After 7 days of coculture, the macrophages were withdrawn and the CRC cells were subsequently collected for further experiments.

### In vivo experimentation

Animal experiments were approved by the Institutional Animal Care and Use Committee of the Sun Yat-sen University and conformed to the “Guide for the Care and Use of Laboratory Animals” of the National Institute of Health in China. NOD-SCID mice were purchased and maintained in pathogen-free conditions at the Experimental Animal Center of Sun Yat-sen University.

For the xenograft tumor model, mice were randomly distributed into five groups. 1 × 10^6^ indicated macrophages were mixed with 5 × 10^6^ SW48 cells and then co-injected subcutaneously into 6-week old NOD-SCID mice. Each group consisted of 5 animals. Tumor development was monitored by digital calipers every four days. The tumor volume was calculated using the following formula: Volume = (Longer diameter × Shorter diameter^2^)/2. After 24 days, the mice were sacrificed and the weight of the tumor was measured. Tumor tissues were harvested and fixed with 10% formalin, followed by hematoxylin and eosin (H&E) and IHC staining.

For the construction of the orthotopic xenograft CRC mouse model, 2 × 10^6^ SW48 cells were mixed with 4 × 10^5^ indicated macrophages which were co-injected into the wall of the cecum in 6-week old NOD-SCID mice. Each group consisted of five mice. After 8 weeks, all the mice were sacrificed. Intestines, livers, and lungs were harvested to assess the tumor burden. Cryosections of the harvested organs were stained using H&E for histological assessment. RNA from the rest of the organs was extracted for qRT-PCR analysis of human hypoxanthine phosphoribosyltransferase (HPRT) mRNA expression.

For cetuximab treatment in nude mouse xenograft tumors, 5 × 10^6^ SW48 cells mixed with or without 1 × 10^6^ Ut-Mφ, KRAS-Mφ, siMOCK-Mφ or siKRAS-Mφ, were injected subcutaneously into 6-week-old NOD-SCID mice. When the tumors reached volumes of 200–300 mm^3^, the mice were randomly assigned to the following two treatments: cetuximab (1 mg twice a week by intraperitoneal injection; *n* = 5 mice per group), or IgG as a control. Tumor growth was monitored every week after cetuximab or IgG treatment.

### Statistical analysis

All data are expressed as mean ± standard deviation (SD) unless otherwise stated. Statistical analysis was performed using SPSS16.0 statistical package. To determine the statistical significance, a two-tailed Student’s *t*-test or one-way ANOVA was used for continuous variables with normal distributions, whereas Mann–Whitney or Kruskal–Wallis test was used when distributions were skewed. Kaplan–Meier plots and log-rank tests were used for survival analysis. A *p-*value of <0.05 was considered to be statistically significant.

## Supplementary information

Supplementary Materials

## Data Availability

Source data for all blot images are provided with this paper. All other relevant data are available from the corresponding author upon reasonable request.
