# Supplementary material for: Mutant KRAS triggers functional reprogramming of tumor-associated macrophages in colorectal cancer
Source: Signal Transduct Target Ther. 2021 Apr 9;6:144. doi: 10.1038/s41392-021-00534-2 (PMC8032794; doi:10.1038/s41392-021-00534-2)

Supplementary Materials for

Mutant KRAS triggers functional reprogramming of tumor-associated macrophages in colorectal cancer

Huashan Liu, Zhenxing Liang, Chi Zhou, Ziwei Zeng, Fengwei Wang, Tuo Hu, Xiaowen He, Xiaojian Wu, Xianrui Wu, Ping Lan
Correspondence to: lanping@mail.sysu.edu.cn or wuxianr5@mail.sysu.edu.cn

This PDF file includes:

Materials and Methods
Figures. S1 to S6
Tables S1 to S4

Other Supplementary Materials for this manuscript include the following:

Source data for immunoblot images

Materials and Methods

Tissue microarray and Immunohistochemistry

338 samples of CRC FFPE tissue were used to construct a tissue microarray (TMA). The invasive front region of tumors was the selected region of interest. Immunohistochemistry (IHC) was performed on TMA and the primary antibodies used were anti-CD163 (1:100, Abcam), anti-CD206 (1:200, Abcam), anti-HIF-1α (1:100, Abcam), anti-GLUT1 (1:100, Abcam), anti-GLUT3 (1:100, Abcam) and anti-HK2 (1:100, Cell Signaling Technology (CST)). TMA was incubated with primary antibodies at 4 °C overnight and then incubated with appropriate secondary antibodies. Afterward, the sections were stained with 3,3'-Diaminobenzidine (DAB) complex and counterstained with haematoxylin. The staining scores were quantified by two independent pathologists who were blinded to the groups, and the scores were based on the staining intensity and proportion of positively stained cells. The staining intensity was divided into the following 4 groups: grade 0 with no staining, grade 1 with weak staining, grade 2 with medium staining and grade 3 with strong staining. The proportion of positively stained cells was determined by the percentage of positive stained area using the following 4 groups: grade 0 = 0, grade 1 = 1-25%, grade 2 = 26-50%, grade 3 = 51-75% and grade 4 = >75%. The staining scores was calculated using the following formula: staining scores = staining intensity × proportion of positively stained cells. Based on this method, the expression levels of HIF-1α, GLUT1, GLUT3 and HK2 was determined by the staining scores on a scale from 0 to 12. The TAM density per patient was determined by counting the number of $CD163^+$ or $CD206^+$ immune cells per tissue

sample using the TMA, as described previously[1].

Immunoblotting

Immunoblotting was performed using anti-KRAS (1:1,000; Novus Biologicals), anti-CD163 (1:1000, Abcam), anti-HLA-DR (1:1000, Abcam), anti-HLA-DR (1:1000, Abcam), anti-iNOS (1:5000, Abcam), anti-E-cadherin (1:1000, CST), anti-vimentin (1:1000, CST), anti-HIF-1α (1:1000, Abcam), anti-hydroxy-HIF-1α (1:1000, Abcam) , anti-PHD2 (1:1000, CST), anti-VHL (1:1000, CST), anti-GAPDH (1:5000, Abcam) and anti-β-actin (1:1000, CST) antibodies (4 °C overnight). Secondary antibodies (1:5000, Abcam) were incubated for 1 h at room temperature. The bolts were visualized with ECL Blotting Detection Reagents (Amersham Biosciences, Little Chalfont, Buckinghamshire, UK).

Immunofluorescence (IF)

Target cells plated on the coverslips (Corning, USA) were fixed with 4% paraformaldehyde for 15 min at room temperature. Afterward, the cells were blocked with 10% bovine serum albumin (BSA, Sigma, Germany) for 1h and incubated with anti-CD68 (1:200, Abcam), followed by an Alexa594-conjugated secondary antibody (Thermo Fisher Scientific, St Peters, MO, USA). For negative-control experiments, isotype matched antibodies were used. After counterstaining with DAPI (Abcam), the slides were imaged using a confocal laser-scanning microscope (Leica TCS-SP8, Leica Microsystems Inc, Buffalo Grove, IL, USA) with a core data acquisition system

(Applied Precision, Bratislava, Slovakia).

Plate colony formation assay

After the indicated treatment, 500 cells were seeded into 6-well plates and cultured at 5% CO_2 at 37° C for 2 weeks. Then the cells were fixed with methanol and stained with 1% crystal violet for 15 min at room temperature. The colony numbers were calculated using Image J software.

Soft agar colony formation assay

After indicated treatment, 500 cells were mixed in 0.3% low melting agarose with DMEM/10% FBS. Afterward, the cells were plated on 0.66% agarose-coated 6-well plates and cultured at 5% CO_2 at 37° C. After 4 weeks, colonies were examined and imaged.

Cell migration and invasion assay

Assays were performed in 24-well plate using an 8-μm pore size chamber (Corning, USA) without Matrigel (for migration assays) or with Matrigel (for invasion assays). 1×10^5 CRC cells with 200μl serum-free cell culture media was placed into the upper chamber, while culture medium supplemented with 10% FBS was placed in the lower chamber. After incubating at 37°C for 48 h, the cells at lower surface of the filters were fixed in methanol for 10 min and stained with crystal violet for 10 min. The cells were then imaged and counted. Values for cell migration or invasion were expressed as the average number of migrated cells in the microscope field in five fields per chamber.

Isolation of RNA and quantitative real-time PCR

Total RNA was extracted from target cells using TRIzol (Invitrogen, CA, USA). The RNA was reverse transcribed to cDNA using a reverse transcription kit (Takara, Otsu, Japan), according to the manufacturer's protocol. Quantitative real-time polymerase chain reaction (qRT-PCR) was performed using a SYBR Green PCR Kit (Takara, Otsu, Japan). Standard curves were generated and the relative amount of target gene mRNA was calculated using the $2^{-\triangle\triangle CT}$ method with normalization to 18S. All primers were obtained from Invitrogen and their specificity was verified by melting curve analysis and agarose gel electrophoresis. The primer sequences are listed were as follows:

Primers used for qRT-PCR analysis.

Genes	Primers	Sequences
β-actin	Forward primer	5′-TCATGAAGTGTGACGTGGACATC-3′
	Reverse primer	5′-CAGGAGGAGCAATGATCTTGATCT-3′
CSF2	Forward primer	5′-GGGAGCATGTGAATGCCATC-3′
	Reverse primer	5′-GGCTCCTGGAGGTCAAACAT-3′
HK2	Forward primer	5′-GATTGCCTCGCATCTGCTTG-3′
	Reverse primer	5′-GCTCCAAGCCCTTTCTCCAT-3′
GLUT1	Forward primer	5′-TGGCATCAACGCTGTCTTCT-3′
	Reverse primer	5′-AACAGCGACACGACAGTGAA-3′
HPRT	Forward primer	5′-TTCCTTGGTCAGGCAGTATAATCC -3′
	Reverse primer	5′-AGTCTGGCTTATATCCAACACTTCG-3′
18S rRNA	Forward primer	5′-CGGCTACCACATCCAAGGAA-3′
	Reverse primer	5′-GCTGGAATTACCGCGGCT-3′

Cell transfection

Transfection of KRAS-specific siRNAs was performed using a pool of four siRNAs (SMART pool siRNA, Dharmacon). KRAS-specific siRNAs (100 nM, siKRAS) or nontargeting control siRNAs (100 nM, siMOCK, Dharmacon) were transfected into

SW620 cells using Dharmafect 4 (Dharmacon) according to the manufacturer's protocol. Transfection of HIF-1α-specific siRNAs in target cells was performed using a heterogeneous mixture of siRNAs (Sigma). To establish SW48 cells stably expressing the $KRASG^{12V}$ mutation, SW48 cells were stably transfected with plasmids encoding either an empty vector (pBabe-puro, Addgene) or $KRASG^{12V}$ (pBabe-puro-$KRAS^{12V}$, Addgene) using the Amaxa system (Lonza). Cells were selected with puromycin (1μg/ml, Merck) for 10 days. Surviving clones were pooled and cultured in DMEM containing 1μg/ml puromycin.

Seahorse XF Extracellular Flux Analysis

The in vitro cell extracellular acidification rate (ECAR) and oxygen consumption rate (OCR) were detected using Seahorse Extracellular Flux Analyzer XF96 (Seahorse Bioscience, North Billerica, MA, USA) according to the manufacturer's instructions. Briefly, 1×10^4 cells per well were seeded in an XF96-well cell culture plate overnight in DMEM containing 10% fetal bovine serum. Then the cells were washed and incubated with bicarbonate-free DMEM with 1mM glutamine for 1 h at 37°C in a CO_2-free incubator. Next, the ECAR and OCR were monitored prior to treatment and after sequential treatment of 10 mM glucose, 1 μM oligomycin, 50 mM 2-deoxy glucose (2-DG), 1 μM carbonyl cyanide 4-(trifluoromethoxy) phenylhydrazone (FCCP) or 0.5μM rotenone/antimycin. Then glycolytic capacity and maximal respiration were calculated as described previously[2].

Flow cytometry analysis

Target cell suspensions were incubated with fluorochrome-conjugated antibodies against CD206 (1:100, eBioscience), HLA-DR (1:100, Biolegend) and their corresponding isotype-matched controls. After incubating for 30 min at room temperature, fluorescent signals were detected and recorded using flow cytometry (BD Biosciences, New Jersey, USA). Raw data were further analyzed and visualized using FlowJo, version 10.0.7.

Glucose consumption and lactate production assays

Glucose consumption and lactate production of target cells were measured using kits from Abcam (ab136955) and BioVision (K607-100), respectively. All procedures were performed according to the manufacturer's recommendations and cell counting was performed using a cell counter.

MTS assays

Target cells (5×10^3 per well) were seeded in 96-well plates. Following 24 h of incubation, the cells were treated with different concentrations of cetuximab (range, 0.01-1.0 mg/ml) for 96 h in triplicate. Cell proliferation was measured with MTS Cell Proliferation Colorimetric Assay Kit (Promega) according to the operating manual.

Flow cytometry for apoptosis identification

Target cells were treated with the indicated concentrations of cetuximab for 96 h and were dissociated using 0.25% trypsin-EDTA, and then collected by centrifugation. The

apoptotic cell death was determined with flow cytometry using an apoptosis detection kit (Multiscience) that stains for annexin V and PI. The percentages of apoptotic cells included the percentages of early (annexin $V^{+}PI^{-}$) and late apoptotic cells (annexin $V^{+}PI^{+}$). Raw data were further analyzed and visualized using FlowJo, version 10.0.7.

Cytokine detection

After undergoing the indicated treatment, cell culture medium of CRC cells was collected, and semi-quantitative analysis of secreted proteins using RayBio Human Cytokine Antibody Array (Raybiotech, Inc. Norcross, GA) was performed according to the manufacturer’s protocol. Cell culture medium of target cells was collected for detection of IL-10, CCL17, TGF-β1, TNF-α, IL-6, IL-1β, CSF2, G-CSF and IL-7 by enzyme-linked immunosorbent assay (ELISA) after indicated treatments. The standard curves were generated using ELISACalc software. All procedures were performed according to the manufacturer’s recommendations.

References

1 Cooks, T. *et al.* Mutant p53 cancers reprogram macrophages to tumor supporting macrophages via exosomal miR-1246. *Nature communications* **9**, 771, doi:10.1038/s41467-018-03224-w (2018).

2 Hu, T. *et al.* Tumor-intrinsic CD47 signal regulates glycolysis and promotes colorectal cancer cell growth and metastasis. *Theranostics* **10**, 4056-4072, doi:10.7150/thno.40860 (2020).

Supplementary Figures

Supplementary Fig. 1

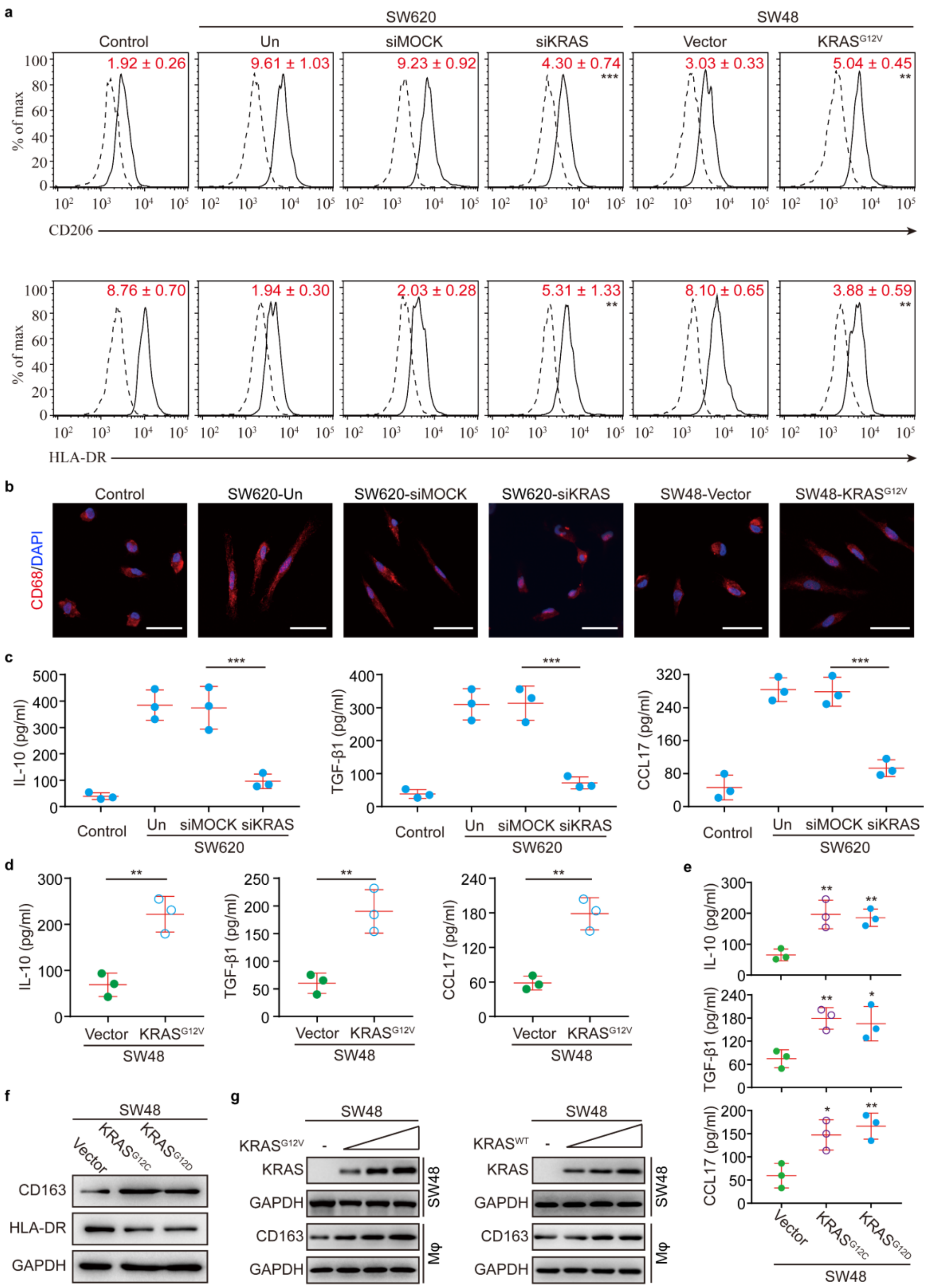

Supplementary Fig. 1 Mutationally activated KRAS in CRC cells directly triggers

functional reprogramming of TAMs. (**a-h**) SW620 cells were untransfected (Un), mock transfected (siMOCK), or transfected with KRAS-siRNAs (siKRAS), and ectopic expression of mutant $KRAS^{G12V}$ or $KRAS^{G12C}$ or $KRAS^{G12D}$ plasmid or an empty vector (Vector) was constructed in SW48 cells. The CM of the indicated cells were harvested to treat macrophages as indicated in **Fig. 2a-c**. (**a**) Representative flow cytometry analysis for CD206/HLA-DR in macrophages. The solid lines represent cells stained with monoclonal antibodies, and dotted lines represent those stained with isotype controls. Numerical values denote the relative mean fluorescence intensity (RelMFI) normalized to isotype controls (mean ± SD) (n = 3). (**b**) Immunofluorescence images of CD68/DAPI in macrophages. Scale bars: 30 μm. (**c-d**) Cytokine levels in the media obtained from macrophage cultures (n = 3). (**e**) Cytokine levels in the media of macrophages treated as indicated (n = 3). (**f**) Representative immunoblot for CD163 and HLA-DR in macrophages treated as indicated. (**g**) SW48 cells were transfected with different concentrations of cDNA encoding wild type KRAS ($KRAS^{WT}$) or $KRAS^{G12V}$ mutation, and then their CM were harvested to treat macrophages as indicated in **Fig. 2a-c**. Immunoblots for KRAS expression levels in SW48 cells treated as indicated and CD163 expression levels in macrophages with each indicated treatment. *P* values are for comparison with "Control" (**a**) or "Vector" (**e**). $*P \leq 0.05$, $**P \leq 0.01$ and $***P \leq 0.001$, by 1-way ANOVA (**a**, **c**, **e**) or 2-tailed Student's t-test (**d**).

Supplementary Fig. 2

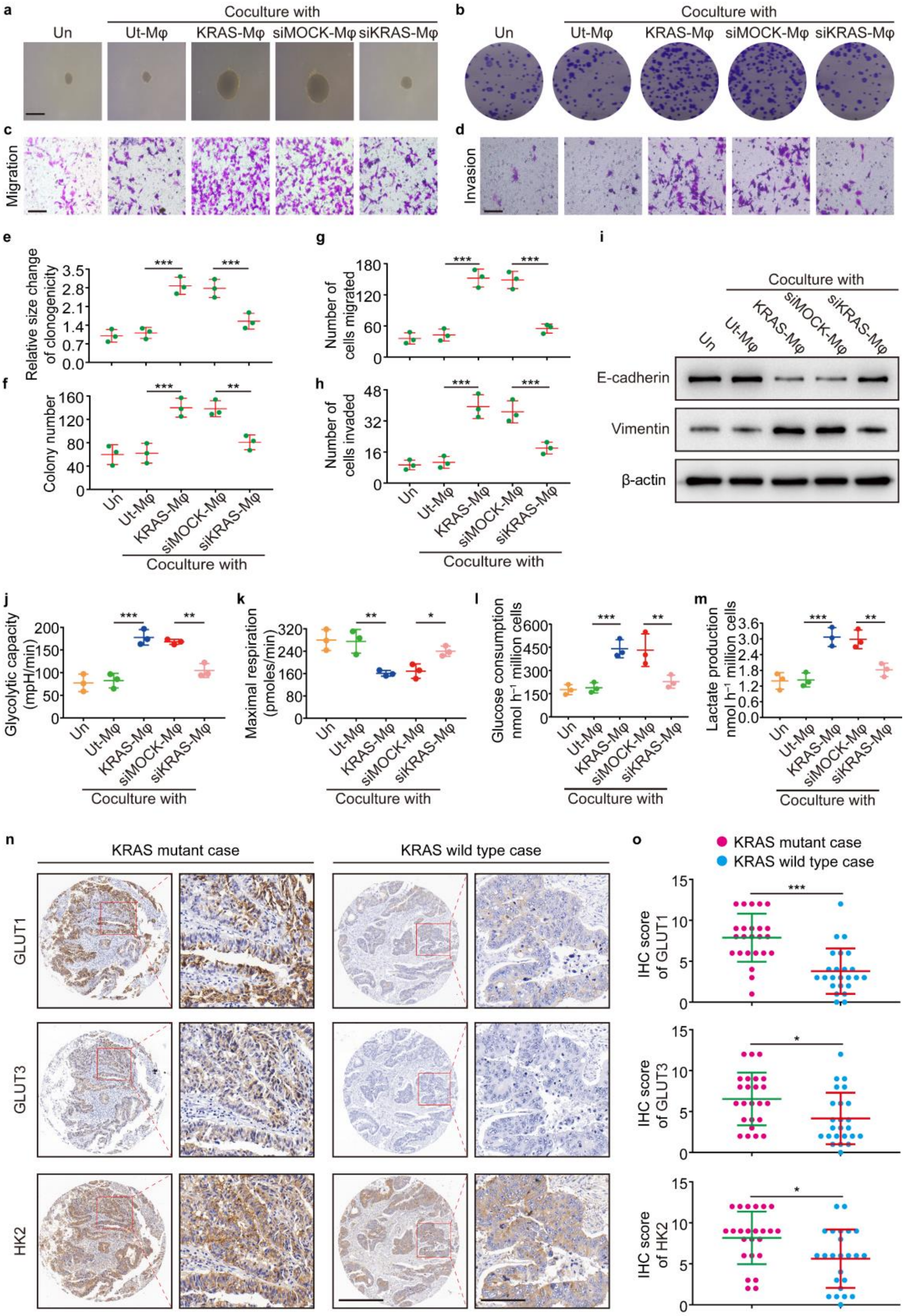

Supplementary Fig. 2 The biological functions of KRAS-reprogrammed macrophages. (**a-m**) KRAS-reprogrammed macrophages (KRAS-Mφ) were obtained

by culture of monocytes in DMEM medium in the presence of 30% CM from KRAS mutant SW620 cells for 6 days. Untreated macrophages (Ut-Mφ) or those exposed to CM from SW620 cells that had been mock transfected (siMOCK-Mφ), or transfected with KRAS-siRNAs (siKRAS-Mφ) were used as controls. SW48 cells were co-cultured with Un-Mφ, KRAS-Mφ, siMOCK-Mφ and siKRAS-Mφ in Transwell systems for 7 days. Subsequently, SW48 cells were collected for the indicated experiments. (**a**) Soft agar colony formation, (**b**) plate colony formation, (**c**) migration and (**d**) invasion assays of SW48 cells that had been treated as indicated. (**e**-**h**) Statistical analysis for panels **a**-**d**. (**i**) Representative immunoblot for the E-cadherin and Vimentin expression in SW48 cells with the indicated treatments. (**j**) Glycolytic capacity, (**k**) maximal respiration, (**l**) glucose consumption, (**m**) lactate production of SW48 cells that had been treated as indicated. (**n**) Epithelial immunostaining of GLUT1, GLUT3 and HK2 in a representative human CRC case carrying mutant KRAS compared to a KRAS wild type case. Scale bars: 400 μm (left), 100 μm (right). (**o**) Quantification of the expression of GLUT1, GLUT3 and HK2 in 24 human CRC samples as described in the Methods section. *$P \leq 0.05$, **$P \leq 0.01$ and ***$P \leq 0.001$, by 1-way ANOVA (**e**-**h**, **j**-**m**) or 2-tailed Student's t-test (**o**).

Supplementary Fig. 3

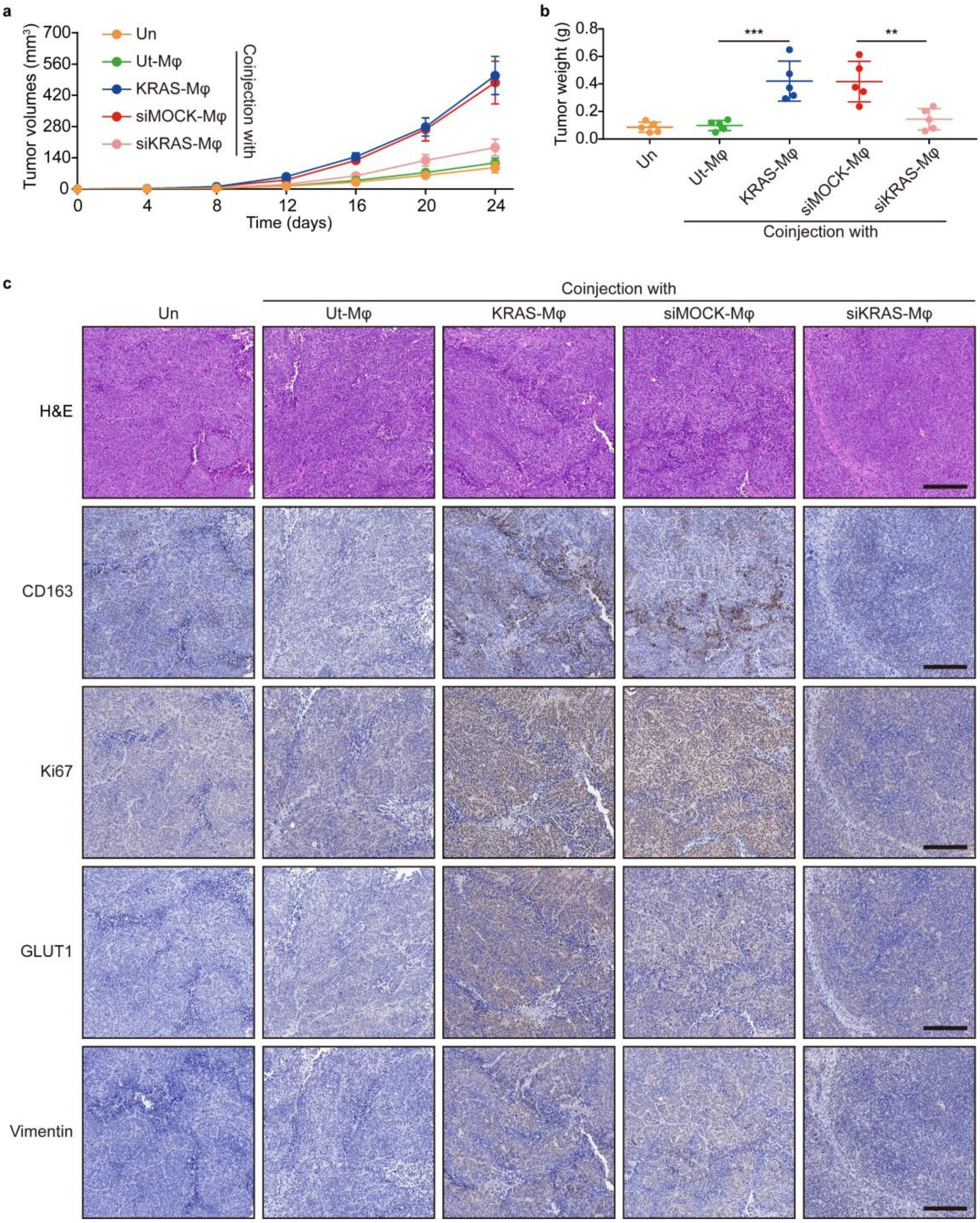

Supplementary Fig. 3 The effects of KRAS-reprogrammed macrophages on CRC cells in subcutaneous xenograft tumors. (**a-c**) KRAS-Mφ were obtained by culture of monocytes in DMEM medium in the presence of 30% CM from KRAS mutant SW620 cells for 6 days. Untreated macrophages (Ut-Mφ) or those exposed to CM from

SW620 cells that had been mock transfected (siMOCK-Mφ), or transfected with KRAS-siRNAs (siKRAS-Mφ) were used as controls. 1×10^6 Ut-Mφ, KRAS-Mφ, siMOCK-Mφ or siKRAS-Mφ were mixed with SW48 cells (5×10^6) and co-injected subcutaneously into 6-week old NOD-SCID mice (n = 5 mice per group). (**a**) Tumor growth curves during the course of each indicated treatment. (**b**) Tumor weights were measured at day 24 in mice xenografted as indicated. (**c**) Immunostaining of CD163, Ki67, GLUT1 and Vimentin in a representative xenograft tumor as described above. Scale bars: 200 μm. Un, SW48 cells alone inoculated into mice (**a-c**). $^{**}P \leq 0.01$ and $^{***}P \leq 0.001$, by 1-way ANOVA (**b**).

Supplementary Fig. 4

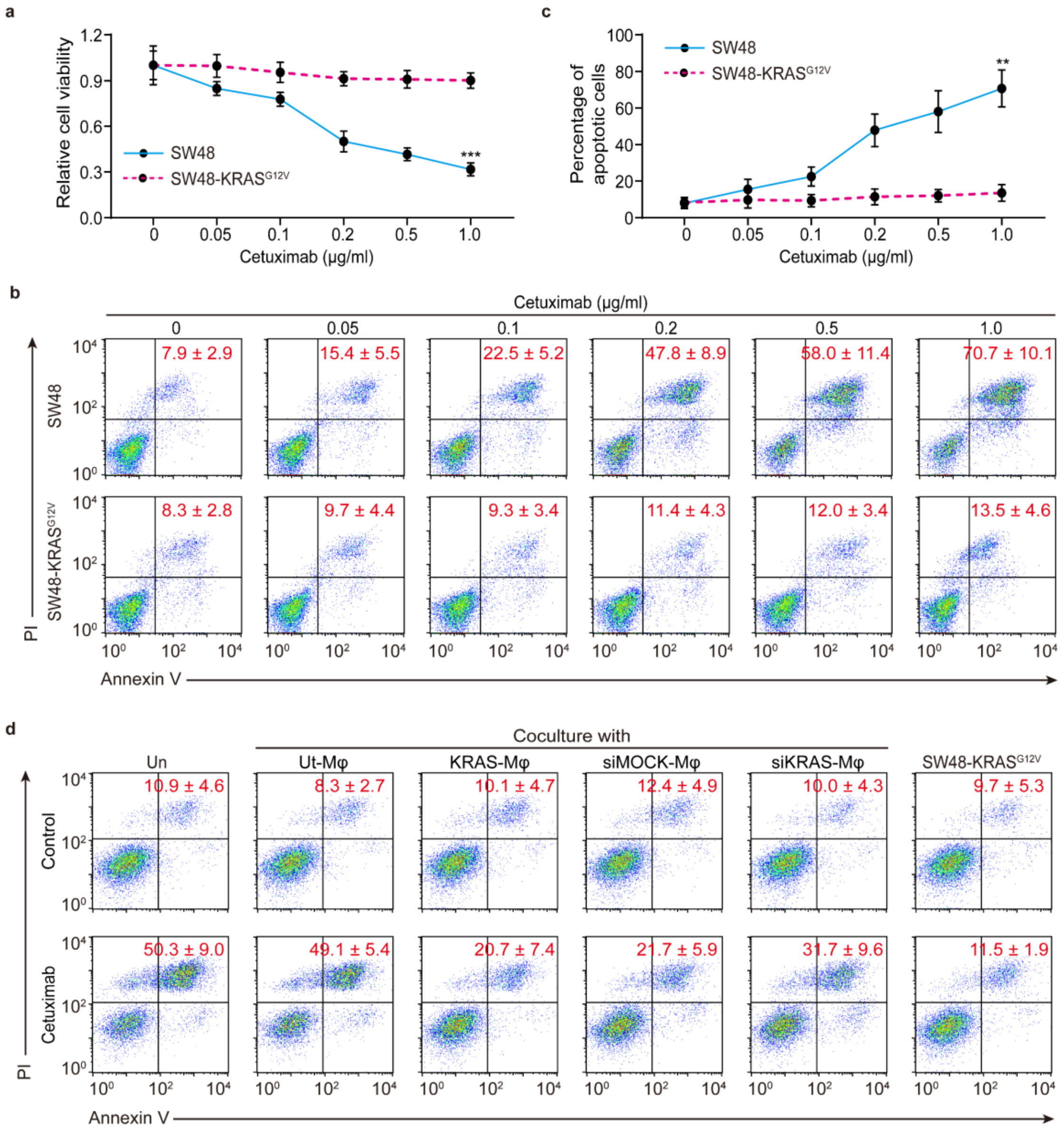

Supplementary Fig. 4 (**a**) SW48 or $KRAS^{G12V}$-transfected SW48 cells were treated with increasing concentrations of cetuximab (0.05-1.0 μg/ml) for 96 hours and evaluated for proliferation by MTS staining (**a**), or for apoptosis by PI/Annexin V staining (**b-c**). (**d**) SW48 cells were co-cultured with the indicated macrophages in Transwell systems for 7 days. Subsequently, the indicated macrophages were treated with 0.2 μg/ml cetuximab or IgG control for 96 hours and evaluated for apoptosis by PI/Annexin V staining. $**P \leq 0.01$ and $***P \leq 0.001$, by 2-tailed Student's t-test (**a**, **c**).

Supplementary Fig. 5

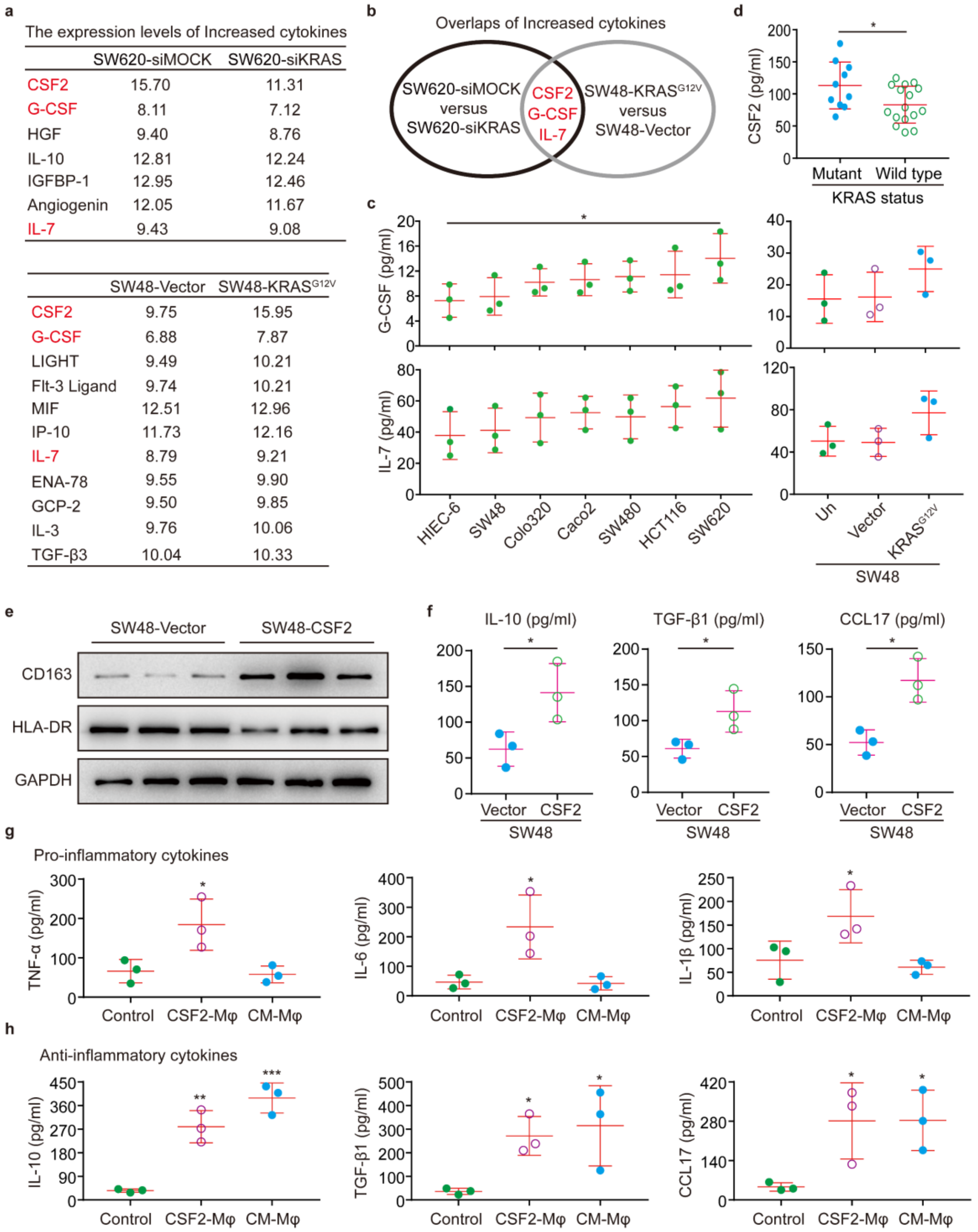

Supplementary Fig. 5 (**a**) A table summarizing the relative signal intensity of indicated cytokines in **Fig. 4a**. (**b**) Venn diagram depicting KRAS-associated proteins from two independent cytokine arrays. (**c**) Levels of G-CSF and IL-7 in the CM of the indicated CRC cells as determined by ELISA assays (n = 3). (**d**) The CSF2 levels by ELISA

assays were compared in the serum of patients between KRAS mutant cases and KRAS wild type cases. (**e-f**) Ectopic expression of CSF2 plasmid or an empty vector (Vector) was constructed in SW48 cells, and then their CM were harvested to treat macrophages as indicated in **Fig. 2a-c**. (**e**) Representative immunoblot for CD163 and HLA-DR in macrophages. (**f**) Cytokine levels in the media obtained from macrophage cultures (n = 3). (**g-h**) Macrophages were obtained by culture of monocytes in culture medium in the presence or absence of 20 ng/ml CSF2 or 30% CM from SW620 cells in 24-well plates for 6 days. Afterward, macrophages were cultured in culture medium for 24 h and cytokine levels in the media were measured by ELISA. *P* values are for comparison with "Control". (**g**) Pro-inflammatory and (**h**) anti-inflammatory cytokine production from CSF2-activated macrophages (CSF2-Mφ) and tumor CM-educated macrophages (CM-Mφ). $^{*}P \leq 0.05$, $^{**}P \leq 0.01$ and $^{***}P \leq 0.001$, by 1-way ANOVA (**c**, **g**-**h**) or 2-tailed Student's t-test (**d**, **f**).

Supplementary Fig. 6

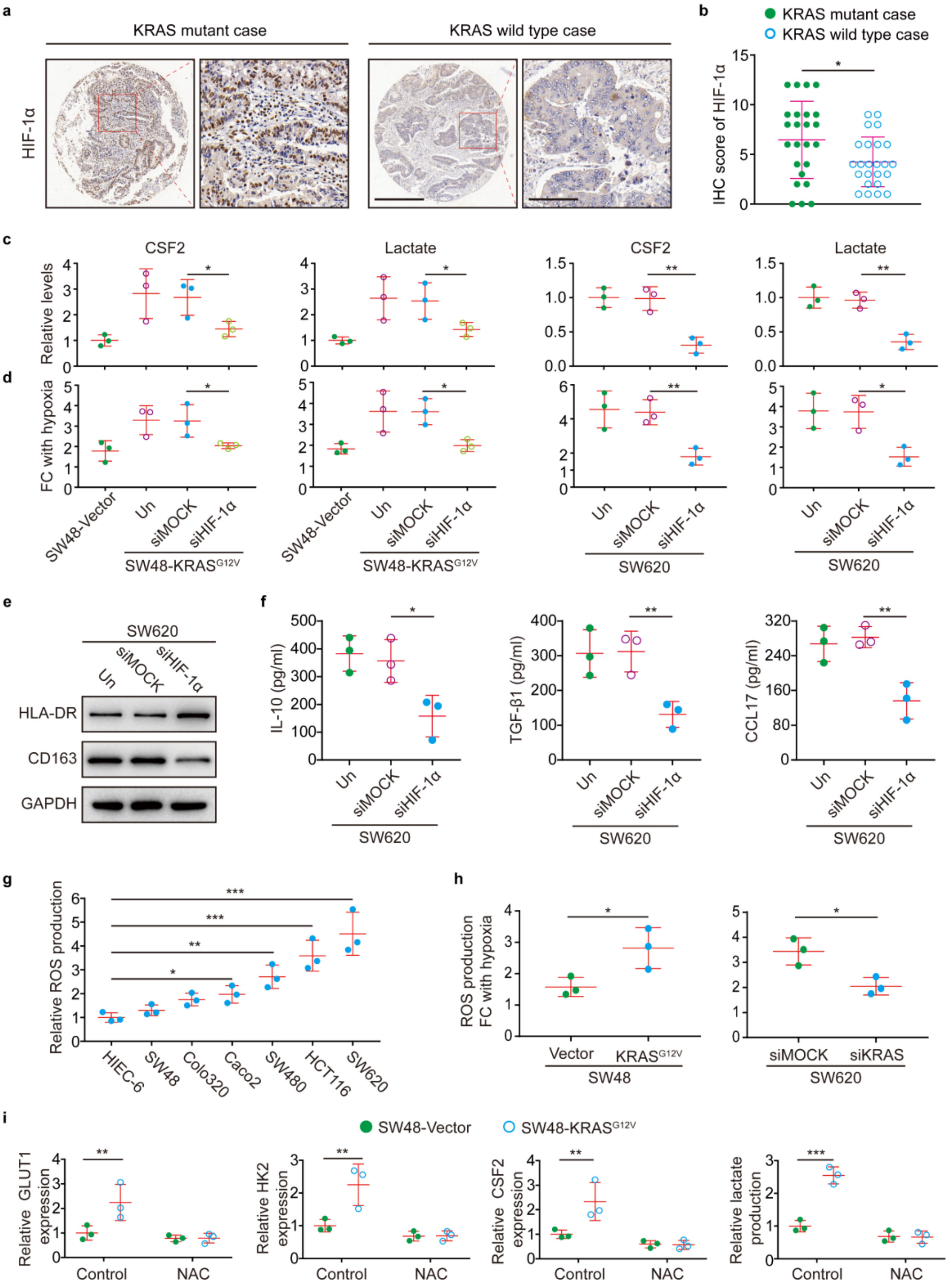

Supplementary Fig. 6 HIF-1α mediates the production of CSF2 and lactate driven by KRAS. (**a**) Immunostaining of HIF-1α in a representative human CRC case carrying mutant KRAS compared to a KRAS wild type case. Scale bars: 400 μm (left), 100 μm

(right). (**b**) Quantification of the expression of HIF-1α in 24 human CRC samples as described in the Methods section. (**c**) The relative levels of CSF2 expression and lactate production of the indicated cells cultured under normoxia (n = 3). (**d**) Fold change (FC) of CSF2 expression or lactate production of the indicated cells in response to hypoxia (n = 3) and the ratio of hypoxic to normoxic gene expression is shown. (**e-f**) SW620 cells were untransfected (Un), mock transfected (siMOCK), or transfected with HIF-1α-siRNAs (siHIF-1α), and then their CM were harvested to treat macrophages as indicated in **Fig. 2a-c**. (**e**) Representative immunoblot for CD163 and HLA-DR in macrophages. (**f**) Cytokine levels in the media obtained from macrophage cultures (n = 3). (**g**) ROS production in the indicated cells (n=3). (**h**) The increase in ROS production with hypoxia was calculated as the FC in ROS in hypoxic cells relative to normoxic controls (n = 3). (**i**) HIF-1α target genes and lactate production were measured by qRT-PCR and a Lactate Assay Kit in Vector or $KRAS^{G12V}$-transfected SW48 (n = 3) that were incubated with 10 mM NAC and cultured under hypoxia (n = 3). β-actin serves as a housekeeping gene for qRT-PCR (**c-d**, **g**). *$P \leq 0.05$, **$P \leq 0.01$ and ***$P \leq 0.001$, by 2-tailed Student's t-test (**b**, **h**, **i**) or 1-way ANOVA (**c-d**, **f-g**).

Table S1. Correlation of TAM density with Clinicopathological features in 338 CRC Patients.

Variables	All cases (n=338)	TAMs determined by CD163 staining			TAMs determined by CD206 staining		
		High TAMs (n=169)	Low TAMs (n=169)	*P* value	High TAMs (n=169)	Low TAMs (n=169)	*P* value
Gender				0.261			0.115
Male	212	101	111		99	113	
Female	126	68	58		70	56	
Age				0.276			0.276
≥60 years	174	92	82		92	82	
< 60 years	164	77	87		77	87	
T stage				< 0.001			< 0.001
T1/T2	105	12	93		14	91	
T3/T4	233	157	76		155	78	
N stage				< 0.001			< 0.001
N0	188	36	152		36	152	
N1/N2	150	133	17		133	17	
M stage				0.002			0.002
M0	328	159	169		159	169	
M1	10	10	0		10	0	
TNM stage				< 0.001			< 0.001
I/II	183	31	152		31	152	
III/IV	155	138	17		138	17	
KRAS status				< 0.001			< 0.001
Mutant	104	77	27		74	30	
Wild type	234	92	142		95	139	

Table S2. Correlation of TAM density with Clinicopathological features in 234 CRC Patients with wild type KRAS.

Variables	TAMs determined by CD163			TAMs determined by CD206		
	High TAMs (n=117)	Low TAMs (n=117)	*P* value	High TAMs (n=117)	Low TAMs (n=117)	*P* value
Gender			1.000			1.000
Male	77	77		77	77	
Female	40	40		40	40	
Age			0.432			0.190
≥ 60 years	65	59		67	57	
< 60 years	52	58		50	60	
T stage			< 0.001			< 0.001
T1/T2	13	75		13	75	
T3/T4	104	42		104	42	
N stage			< 0.001			< 0.001
N0	55	117		55	117	
N1/N2	62	0		62	0	
M stage			0.498			0.498
M0	115	117		115	117	
M1	2	0		2	0	
TNM stage			< 0.001			< 0.001
I/II	53	117		53	117	
III/IV	64	0		64	0	

Table S3. Correlation of TAM density with Clinicopathological features in 104 CRC Patients with mutant KRAS.

Variables	TAMs determined by CD163			TAMs determined by CD206		
	High TAMs (n=52)	Low TAMs (n=52)	*P* value	High TAMs (n=52)	Low TAMs (n=52)	*P* value
Gender			1.000			0.114
Male	29	29		25	33	
Female	23	23		27	19	
Age			0.239			0.050
≥ 60 years	28	22		30	20	
< 60 years	24	30		22	32	
T stage			0.001			< 0.001
T1/T2	2	15		1	16	
T3/T4	50	37		51	36	
N stage			0.014			0.013
N0	3	13		3	13	
N1/N2	49	39		49	39	
M stage			0.006			0.006
M0	44	52		44	52	
M1	8	0		8	0	
TNM stage			< 0.001			< 0.001
I/II	0	13		0	13	
III/IV	52	39		52	39	

Table S4. Correlation of KRAS status with Clinicopathological features in 338 CRC Patients.

Variables	All cases (n=338)	Patients with mutant KRAS (n=104)	Patients with wild type KRAS (n=234)	*P* value
Gender				0.078
Male	212	58	154	
Female	126	46	80	
Age				0.404
≥ 60 years	174	50	124	
< 60 years	164	54	110	
CEA (ng/ml)				0.081
≥ 5	120	44	76	
< 5	218	60	158	
T stage				< 0.001
T1/T2	105	17	88	
T3/T4	233	87	146	
N stage				< 0.001
N0	188	16	172	
N1/N2	150	88	62	
M stage				0.002
M0	328	96	232	
M1	10	8	2	
TNM stage				< 0.001
I/II	183	13	170	
III/IV	155	91	64	

Source data for immunoblot images

Full unedited blot for Fig.2e

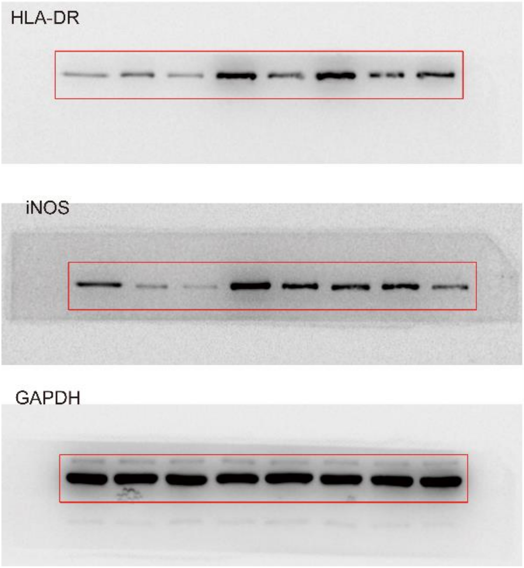

Full unedited blot for Fig.6a

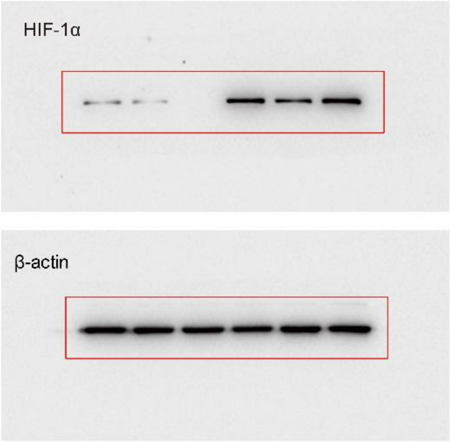

Full unedited blot for Fig.6b

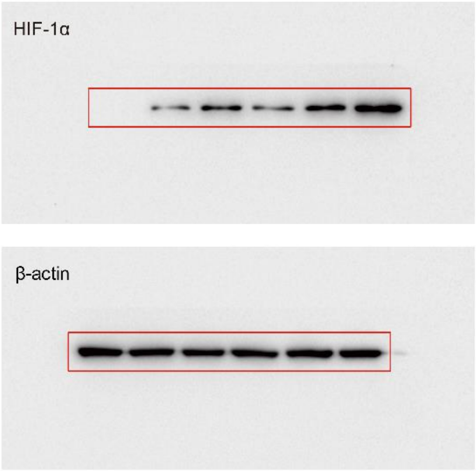

Full unedited blot for Fig.6c

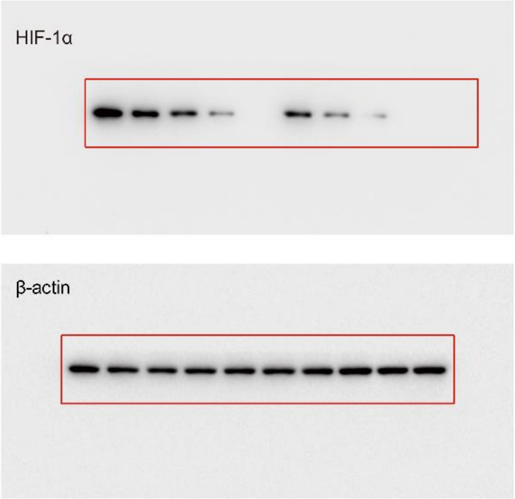

Full unedited blot for Fig.6f

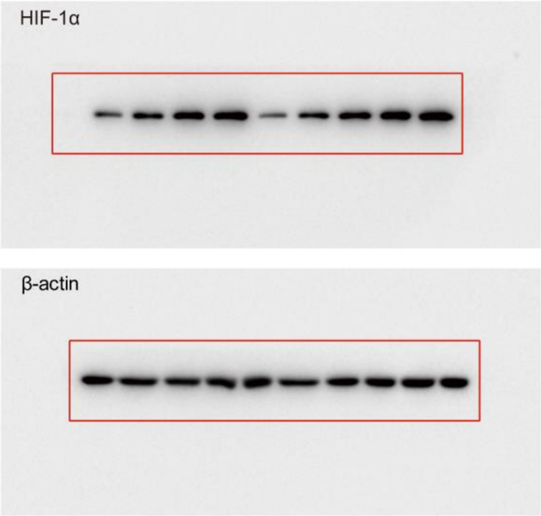

Full unedited blot for Fig.6i

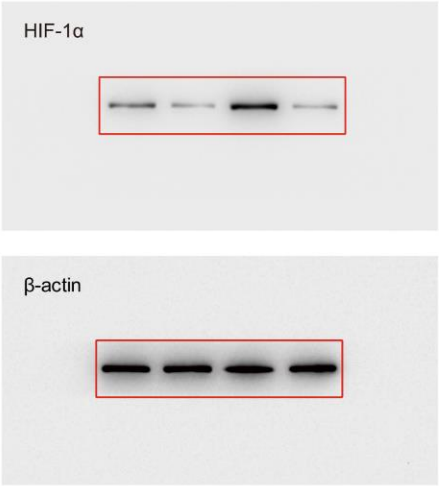

Full unedited blot for Fig.6e

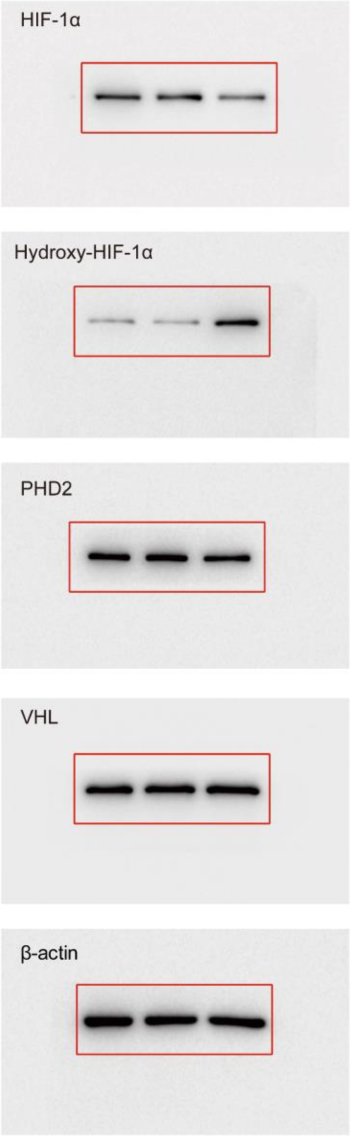

Full unedited blot for Fig.6j

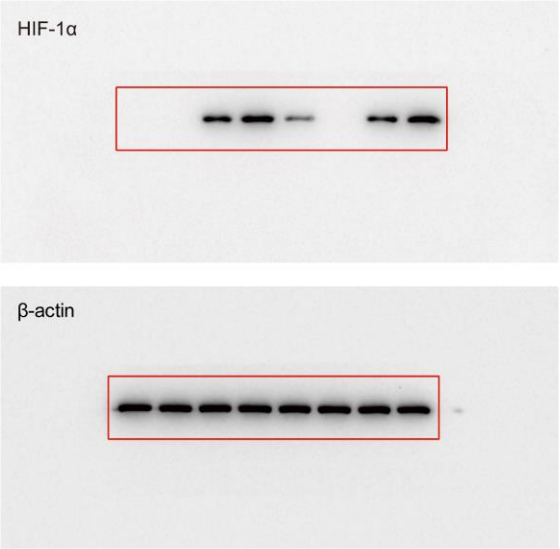

Full unedited blot for Supplementary Fig.5e

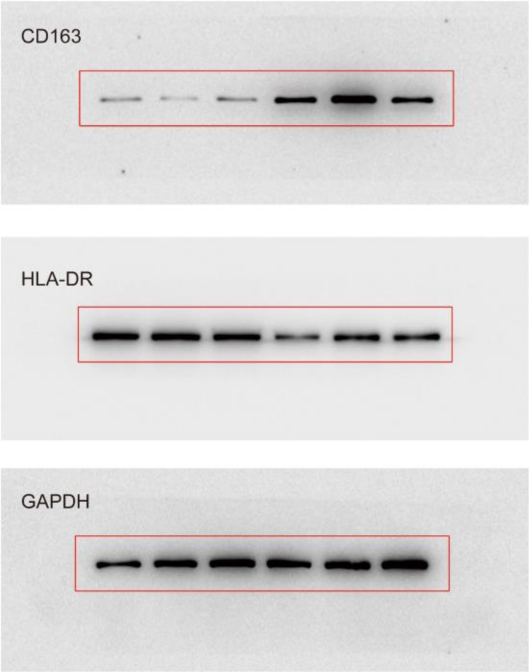

Full unedited blot for Supplementary Fig.1f

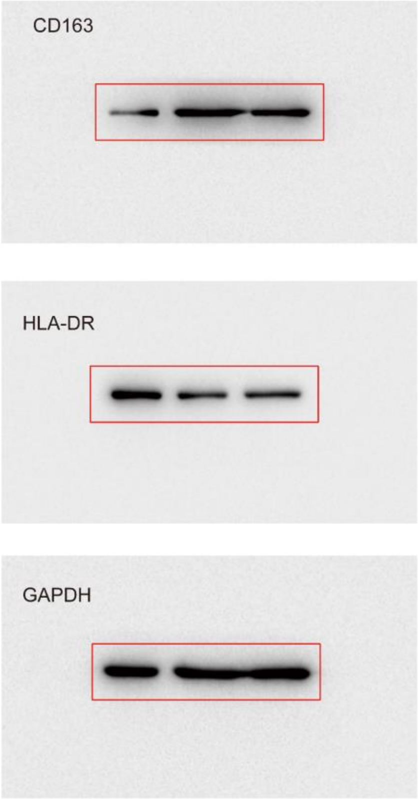

Full unedited blot for Supplementary Fig.2i

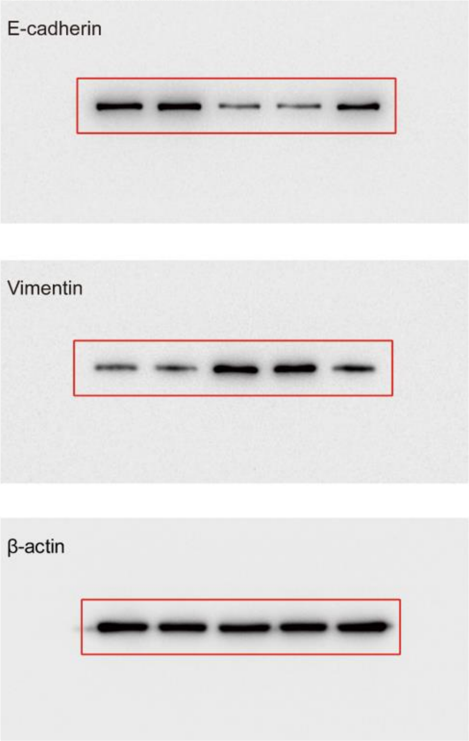

Full unedited blot for Supplementary Fig.6e

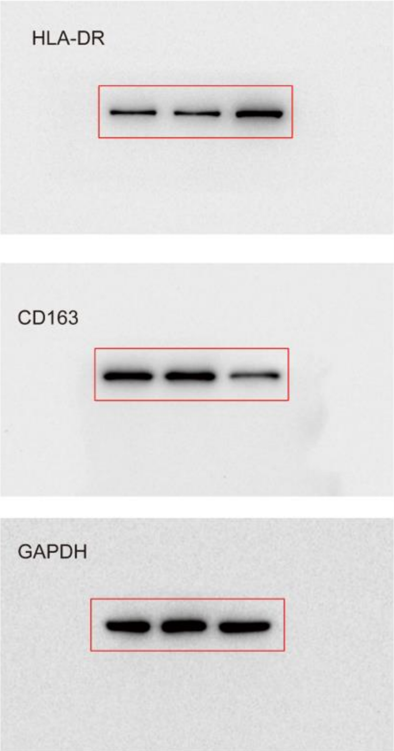

Full unedited blot for Supplementary Fig.1g

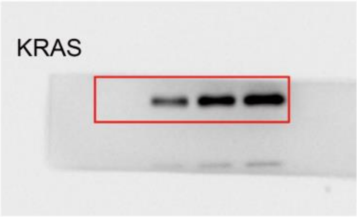

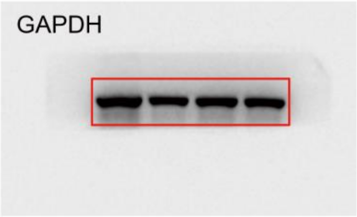

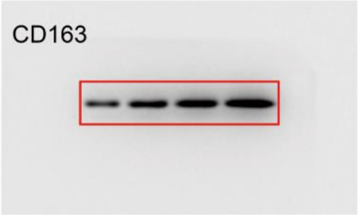

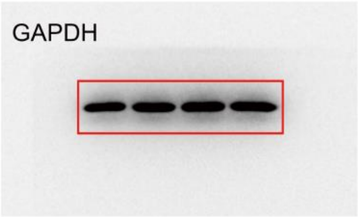

Full unedited blot for Supplementary Fig.1g

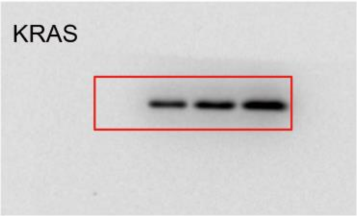

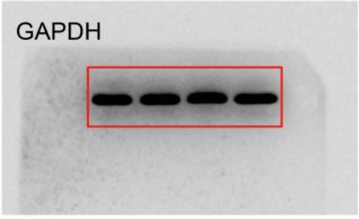

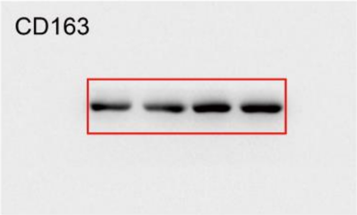

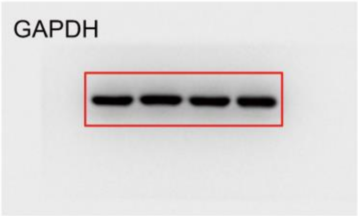

Supplement: Supplementary file 1 — Supplementary Materials [file 41392_2021_534_MOESM1_ESM.pdf]
